# Albumin Neutralizes Hydrophobic Toxins and Modulates *Candida albicans* Pathogenicity

**DOI:** 10.1128/mBio.00531-21

**Published:** 2021-06-22

**Authors:** Sophie Austermeier, Marina Pekmezović, Pauline Porschitz, Sejeong Lee, Nessim Kichik, David L. Moyes, Jemima Ho, Natalia K. Kotowicz, Julian R. Naglik, Bernhard Hube, Mark S. Gresnigt

**Affiliations:** a Department of Microbial Pathogenicity Mechanisms, Leibniz Institute for Natural Product Research and Infection Biology, Hans Knöll Institute, Jena, Germany; b Centre for Host-Microbiome Interactions, Faculty of Dentistry, Oral and Craniofacial Sciences, King’s College London, London, United Kingdom; c Institute for Microbiology, Friedrich Schiller University of Jena, Jena, Germany; d Junior Research Group Adaptive Pathogenicity Strategies, Leibniz Institute for Natural Product Research and Infection Biology, Hans Knöll Institute, Jena, Germany; University of Toronto

**Keywords:** microbial cytotoxicity, toxins, serum proteins, cytolysis, virulence

## Abstract

Albumin is abundant in serum but is also excreted at mucosal surfaces and enters tissues when inflammation increases vascular permeability. Host-associated opportunistic pathogens encounter albumin during commensalism and when causing infections. Considering the ubiquitous presence of albumin, we investigated its role in the pathogenesis of infections with the model human fungal pathogen, Candida albicans. Albumin was introduced in various *in vitro* models that mimic different stages of systemic or mucosal candidiasis, where it reduced the ability of C. albicans to damage host cells. The amphipathic toxin candidalysin mediates necrotic host cell damage induced by C. albicans. Using cellular and biophysical assays, we determined that albumin functions by neutralizing candidalysin through hydrophobic interactions. We discovered that albumin, similarly, can neutralize a variety of fungal (α-amanitin), bacterial (streptolysin O and staurosporin), and insect (melittin) hydrophobic toxins. These data suggest albumin as a defense mechanism against toxins, which can play a role in the pathogenesis of microbial infections.

## INTRODUCTION

Human albumin is exclusively produced in the liver (1) and has three essential functions: maintenance of oncotic pressure (1), acting as a shuttle for hydrophobic molecules such as fatty acids (2), and possessing antioxidative properties (3). Around 40% of the total albumin is located intravascularly as serum albumin, whereas 60% can be found in the interstitial space (1). Serum albumin is also excreted into the intestine (4) and exits the bloodstream when the vasculature is permeabilized during acute inflammation (5). Therefore, mucosal microbes of the human microbiota, as well as microbial pathogens causing mucosal or systemic infections, are in constant contact with different albumin concentrations.

One of the most common fungal members of the human microbiota and a frequent cause of mucosal and systemic infections is the yeast Candida albicans. In health, C. albicans colonizes the human gut, oral cavity, or vaginal tract of most individuals as a harmless commensal (6–9). However, C. albicans is also an opportunistic pathogen that can cause severe mucosal and systemic infections in predisposed hosts (10). As such, individuals with attenuated immunity, a disturbed microbiota, or a disrupted intestinal barrier are at risk of life-threatening invasive candidiasis (11–13).

These predisposing factors permit C. albicans translocation through the intestinal epithelial barrier, resulting in an invasion of the bloodstream and dissemination to vital organs such as the kidney or liver (14–16). During the pathogenesis of systemic candidiasis, blood is an important and unique environment, harboring various stimuli and threats for the fungus. Many studies have demonstrated the interaction of C. albicans with coagulation factors (17, 18), immune mediators such as complement proteins (19, 20), and immune cells present in blood (21, 22). Human serum is also able to modulate the pathogenicity of C. albicans (23–25). For example, serum is a potent inducer of hypha formation, the invasive morphology of C. albicans (26), but it surprisingly also reduces C. albicans-induced damage to host cells (25). Despite this, the molecular mechanisms by which serum modulates C. albicans pathogenicity remain unclear. Furthermore, no systematic approaches have been undertaken to elucidate the role of individual proteins mediating the antifungal effects of serum. In this context, even the most abundant serum protein, albumin, has remained largely unexplored in relation to its role during C. albicans host-pathogen interactions.

Considering the abundant presence of albumin in the human body, we investigated whether albumin plays a role during microbial pathogenesis, focusing on C. albicans as a model mucosal pathogen. Using *in vitro* and *in vivo* infection models and cellular and biophysical assays, albumin was found to modulate C. albicans pathogenicity by neutralizing the peptide toxin candidalysin via hydrophobic interactions. Importantly, albumin also neutralized a variety of bacterial, fungal, and insect hydrophobic toxins, providing an important biological mechanism by which albumin may protect host organisms against toxin activity.

## RESULTS

### Human albumin reduces *C. albicans* pathogenicity in an intestinal translocation model.

Systemic candidiasis often originates from the translocation of C. albicans across the intestinal tract, where the fungus normally resides as a commensal (16). To dissect the influence of albumin on C. albicans during its interaction with intestinal epithelial cells (IECs), albumin was introduced in an intestinal infection model that can be used to study various pathogenicity mechanisms of C. albicans and its potential to translocate through intestinal barriers (27, 28). In this transwell system, albumin was introduced to the basal side of the epithelial barrier.

**FIG 1:**
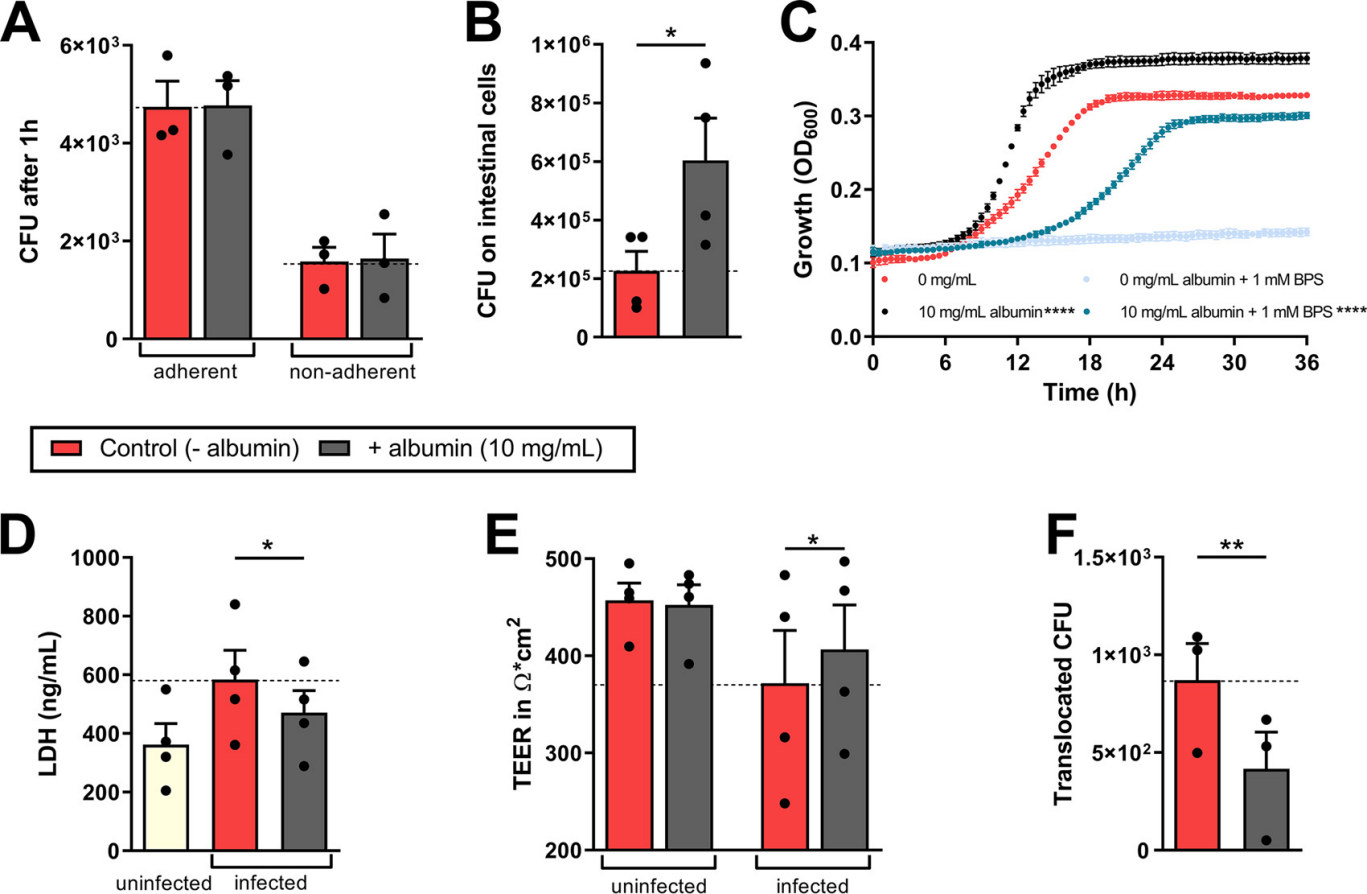
Influence of albumin on C. albicans pathogenicity mechanisms. (A) Adherent and nonadherent CFU of C. albicans to intestinal epithelial cells after 1 h in the presence or absence of 5 mg/ml albumin (HSA) in the lower compartment. (B) Fungal CFU grown on the intestinal epithelial cells were quantified after 18 h of infection with C. albicans in the presence or absence of 5 mg/ml albumin in the lower compartment. (C) Proliferation of C. albicans in the presence of 0 or 10 mg/ml albumin and of 0 or 1 mM BPS in RPMI was measured by determining the OD_600_ at 37°C for 36 h. (D to F) After 18 h of infection, the LDH release of intestinal epithelial cells in ng/ml was quantified from a 96-well plate (D), and the TEER (E) and CFU translocated through the intestinal epithelial layer (F) were quantified from the transwell assay. Bars represent mean values and the SEM of *n* = 3 (A, C, and F) or *n* = 4 (B, D, and E) independent experiments. Significances were calculated using a paired, parametric, two-tailed Student *t* test and compared to the control (= 0 mg/ml albumin or for panel B; additionally, 0 mg/ml + BPS versus 10 mg/ml + BPS) (*, *P* ≤ 0.05; **, *P* ≤ 0.01; ****, *P* ≤ 0.0001).

C. albicans adhesion to the intestinal epithelial barrier was unaffected by albumin (Fig. 1A), but fungal burdens increased (Fig. 1B). This growth-promoting effect was validated in growth curve experiments, where C. albicans was exposed to albumin (Fig. 1C). Given that albumin may serve as an iron source for C. albicans, by improving heme availability (29), we assessed C. albicans growth in the presence of the iron-chelating agent bathophenanthroline sulfonate (BPS). In line with previous findings (29), BPS inhibited C. albicans growth, while the addition of albumin rescued fungal proliferation (Fig. 1C). However, albumin-induced fungal growth did not correlate with enhanced host cell damage; rather albumin significantly reduced C. albicans*-*induced necrotic cell death of IECs (Fig. 1D). Consistent with reduced host cell damage, albumin also rescued IEC barrier integrity as assessed by transepithelial electrical resistance (TEER) (Fig. 1E). Likewise, fungal translocation across the IEC barrier decreased significantly in the presence of albumin in the basal compartment of the model (Fig. 1F).

Collectively, the data suggest that albumin supports C. albicans growth, at least partially by providing iron, but reduces fungal pathogenicity by reducing epithelial damage and translocation of C. albicans across IECs.

### The protective effect of albumin against *C. albicans* is host cell type independent.

**FIG 2:**
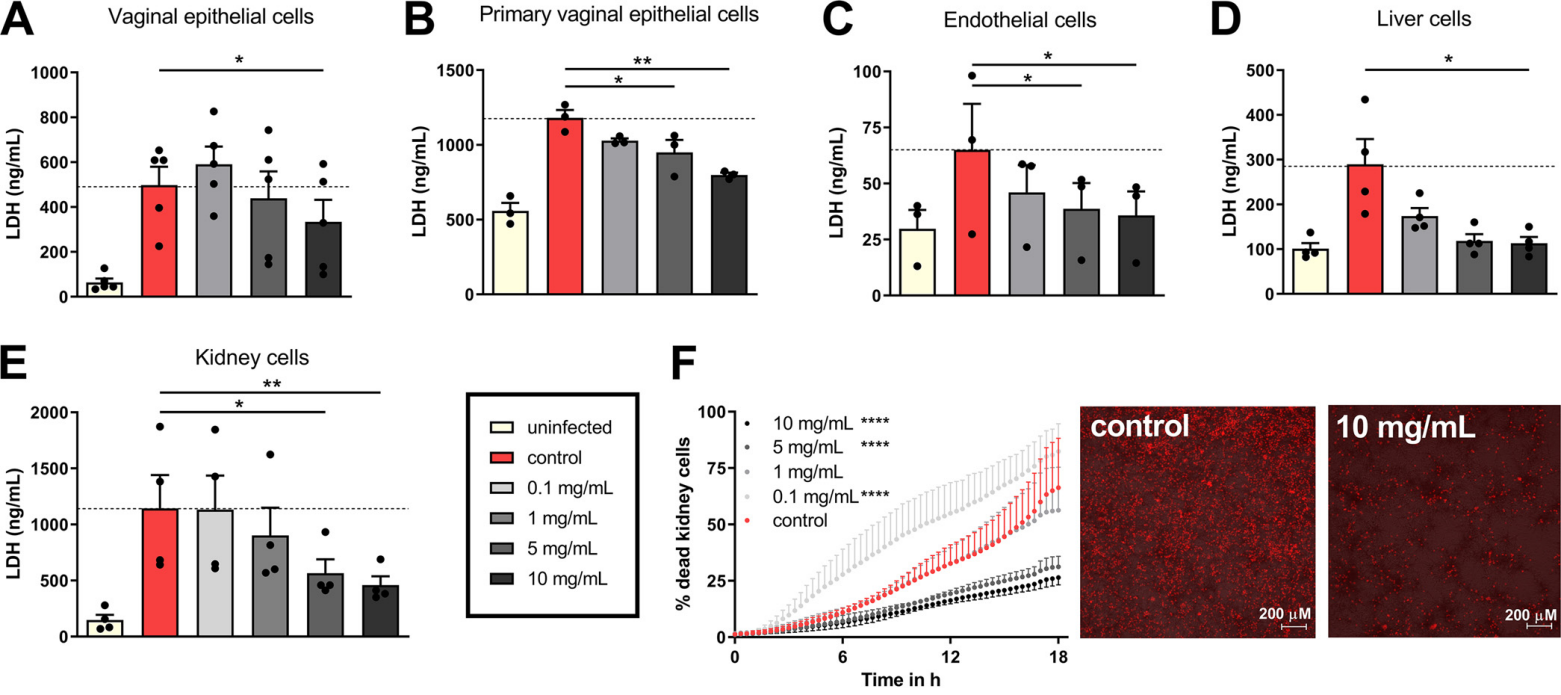
Influence of albumin on C. albicans-induced damage to different host cells. LDH release in ng/ml of vaginal epithelial (A-431) (A), primary vaginal epithelial cells (B), human umbilical vein endothelial cells (HUVECs) (C), liver (HepaRG) (D), and kidney cells (HEK293A) (E) after 18 h of infection with C. albicans in the presence of 0 to 10 mg/ml albumin. (F) Percentage of dead kidney cells infected with C. albicans in the presence of 0 to 10 mg/ml albumin over 18 h. Cell death dynamics over time were quantified using PI staining. Representative microscopy images show the PI signal of kidney cells infected with C. albicans in the presence or absence of 10 mg/ml albumin at 18 h postinfection. Red staining represents necrotic cells that lost their membrane integrity and intercalated the PI dye in their DNA. Bars represent mean values and SEM of *n* = 3 (B, C, and F), *n* = 4 (D and E), or *n* = 5 (A) independent experiments. A one-way ANOVA with a Dunnett’s posttest correction was used to determine statistical significance compared to the control (= 0 mg/ml albumin) (*, *P* ≤ 0.05; **, *P* ≤ 0.01; ****, *P* ≤ 0.0001).

To determine whether the protective effect of albumin was exclusive to intestinal epithelial cells, the influence of albumin on C. albicans-induced damage was also investigated on other biologically relevant host cells. During mucosal and systemic infections, C. albicans interacts with various tissues, including vaginal epithelial (modeled by A-431 cell line and primary human vaginal cells), endothelial (modeled by primary human umbilical cord vascular endothelial cells [HUVECs]), liver (modeled by immortalized liver cells [HepaRG]), and kidney (modeled by human embryonic kidney cells [HEK293A]) cells. Albumin reduced C. albicans*-*induced damage to all these host cell types in a dose-dependent fashion (Fig. 2A to E).

This suggests that the protective effect of albumin against C. albicans-induced damage is universal and independent of the host cell type. Therefore, elucidating the mechanism of albumin protection was prioritized.

### Albumin protects kidney cells against *C. albicans*-induced damage.

**FIG 3:**
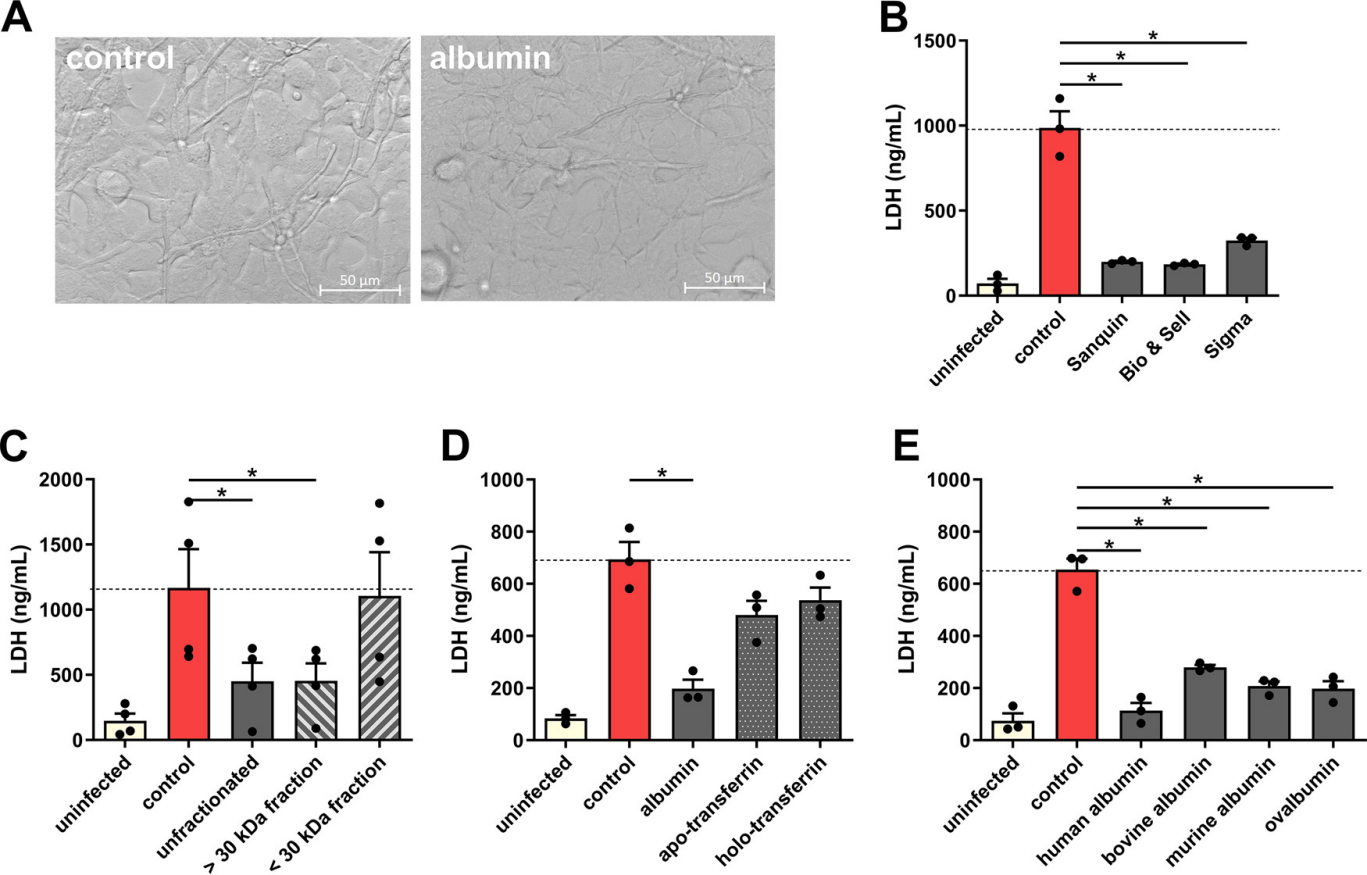
The damage-reducing effect on C. albicans-induced host cell damage is albumin specific. (A) Representative microscopy images (40× magnification) of C. albicans-infected kidney cells in the presence or absence of human albumin (Sanquin Plasma Product B.V.) at 5.5 h postinfection. (B to E) Damage of kidney cells measured by LDH release after 18 h in the presence of 10 mg/ml human albumin solutions of three different manufacturers (Sanquin Plasma Products B.V., Albuman; Bio & Sell catalog number HSA.FV.0025; Sigma, catalog number A6909) in comparison to the control without albumin (B); the presence of 10 mg/ml albumin or albumin, which was fractionated by molecular weight filters with a cutoff 30 kDa, both the >30-kDa and the <30-kDa fractions are shown (C); the presence of 10 mg/ml albumin, apo-transferrin or holo-transferrin (D); and for the presence of 10 mg/ml human (Sanquin Plasma Products B.V., Albuman), bovine, murine, or ovalbumin (E) were tested. Bars represent mean values and the SEM of *n* = 3 (B, D, and E) or *n* = 4 (C) independent experiments. Significances were calculated using a paired, parametric, two-tailed Student *t* test (*, *P* ≤ 0.05).

Human embryonic kidney cells were used to study the effect of albumin on C. albicans-induced damage in more detail. To validate that necrotic cell death (Fig. 2E) is affected by albumin, propidium iodide (PI) influx, as a proxy for the loss of membrane integrity associated with necrosis, was assessed. Similar to reduced lactate dehydrogenase (LDH) release, albumin significantly reduced PI influx into kidney cells over time (Fig. 2F; see also Movies S1 and S2 in the supplemental material), confirming that albumin inhibits C. albicans-induced necrotic cell damage. No drastic differences in fungal morphology were observed during the infection of kidney cells in the presence of albumin (Fig. 3A). Albumin from three different suppliers reduced the ability of C. albicans to damage kidney cells (Fig. 3B), ruling out a nonspecific effect originating from the albumin preparation. Likewise, the influence of small-molecule preservatives in the albumin preparations was excluded using molecular-weight-cutoff filters, showing that only fractions with a molecular weights above 30 kDa, including 66.4-kDa albumin (1), prevent C. albicans*-*induced damage (Fig. 3C). Furthermore, the effect was specific to albumin, since other human serum proteins, namely, apo-transferrin and holo-transferrin, did not significantly reduce C. albicans-induced damage (Fig. 3D), whereas bovine, murine, and chicken (ovalbumin) albumin were all able to reduce C. albicans-induced damage (Fig. 3E).

10.1128/mBio.00531-21.1 MOVIE S1 PI staining of kidney cells infected with C. albicans. Necrotic kidney cells are stained in red by PI. Infection was imaged over 18 h, and microscopic pictures were taken every 20 min using Zeiss Cell Discoverer 7. Download Movie S1, AVI file, 1.0 MB. Copyright © 2021 Austermeier et al. 2021 Austermeier et al. https://creativecommons.org/licenses/by/4.0/ This content is distributed under the terms of the Creative Commons Attribution 4.0 International license.

10.1128/mBio.00531-21.2 MOVIE S2 PI staining of kidney cells infected with C. albicans in the presence of albumin. Necrotic kidney cells are stained in red by PI. Infection was imaged over 18 h, and microscopic pictures were taken every 20 min using Zeiss Cell Discoverer 7. Download Movie S2, AVI file, 0.8 MB. Copyright © 2021 Austermeier et al. 2021 Austermeier et al. https://creativecommons.org/licenses/by/4.0/ This content is distributed under the terms of the Creative Commons Attribution 4.0 International license.

**FIG 4:**
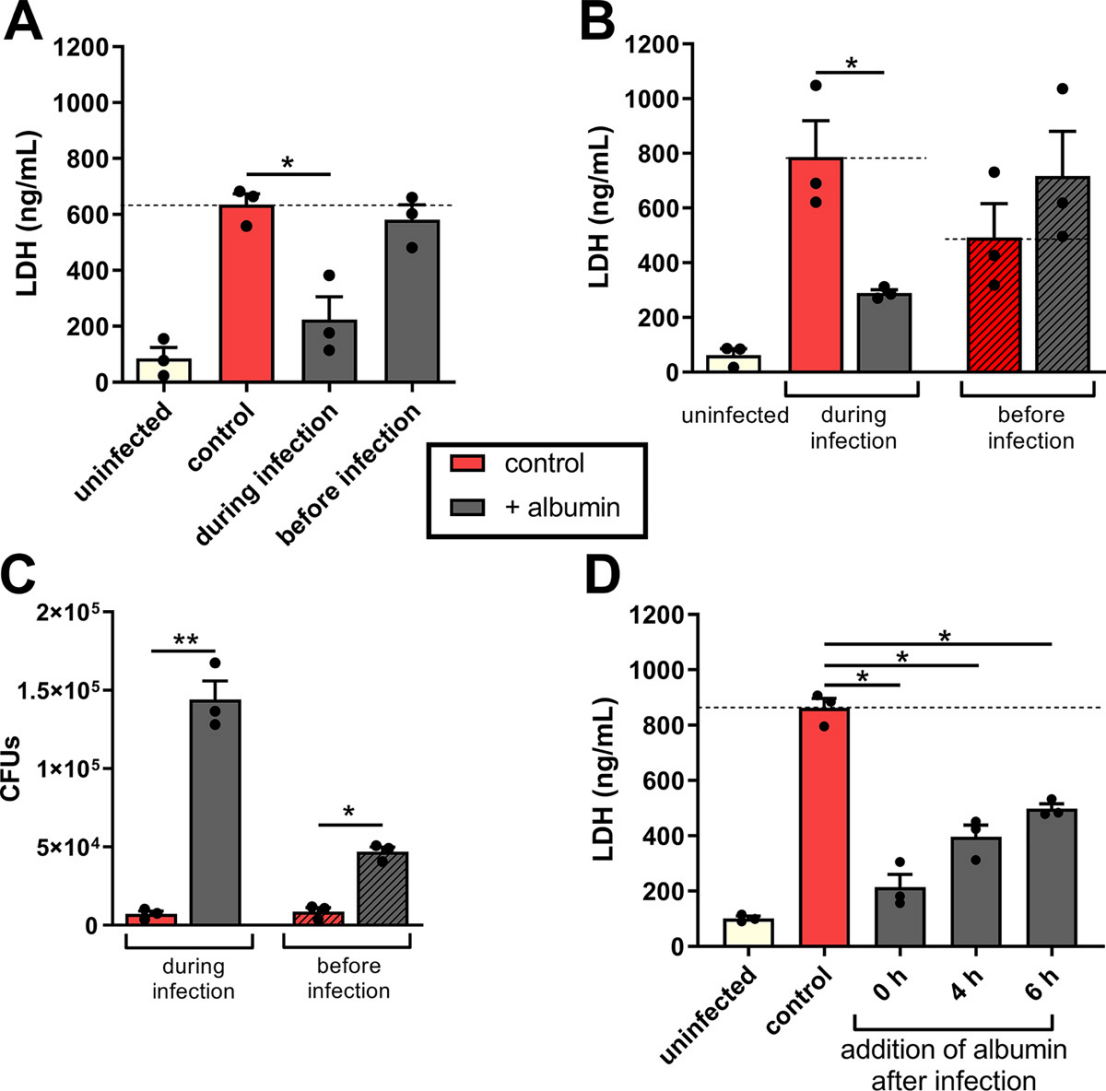
The presence of albumin during infection is essential for its protective effect. (A) Kidney cells were infected with C. albicans without albumin, with albumin present during infection, or with albumin present in the kidney cells medium only before the infection, and the LDH release was measured after 18 h. (B) Kidney cells were infected with C. albicans without albumin, with albumin present during infection, or with C. albicans that were preincubated with or without 10 mg/ml albumin for 1 h before infection, followed by removal of the protein. (C) CFU of C. albicans grown on the kidney cells conditioned in panel B. (D) LDH release of kidney cells that were infected with C. albicans, and albumin was added 0, 4, or 6 h after infection. Bars represent mean values and the SEM of *n* = 3 independent experiments. Significances were calculated using a paired, parametric, two-tailed Student *t* test (*, *P* ≤ 0.05; **, *P* ≤ 0.01).

Further analysis showed that 1 h of preincubation of kidney cells alone with albumin (followed by its removal) (Fig. 4A) prior to infection did not affect the ability of C. albicans to damage kidney cells. Preincubation of the fungus 1 h prior to infection with albumin (followed by its removal) also did not neutralize the fungal damage capacity to kidney cells (Fig. 4B). Rather, the preincubation of C. albicans with albumin increased its ability to damage kidney cells, presumably due to its growth-promoting effect on C. albicans (Fig. 4C, right panel). In addition, we questioned whether albumin is still protective after C. albicans hyphae penetrated host tissue and formed invasion pockets. The protective effect of albumin was decreased when albumin was added 4 or 6 h after infection (Fig. 4D). Collectively, these results strongly suggest that albumin needs to be present during infection in order to prevent C. albicans-induced damage to host cells.

### Albumin neutralizes the *C. albicans* peptide toxin candidalysin through hydrophobic interactions.

**FIG 5:**
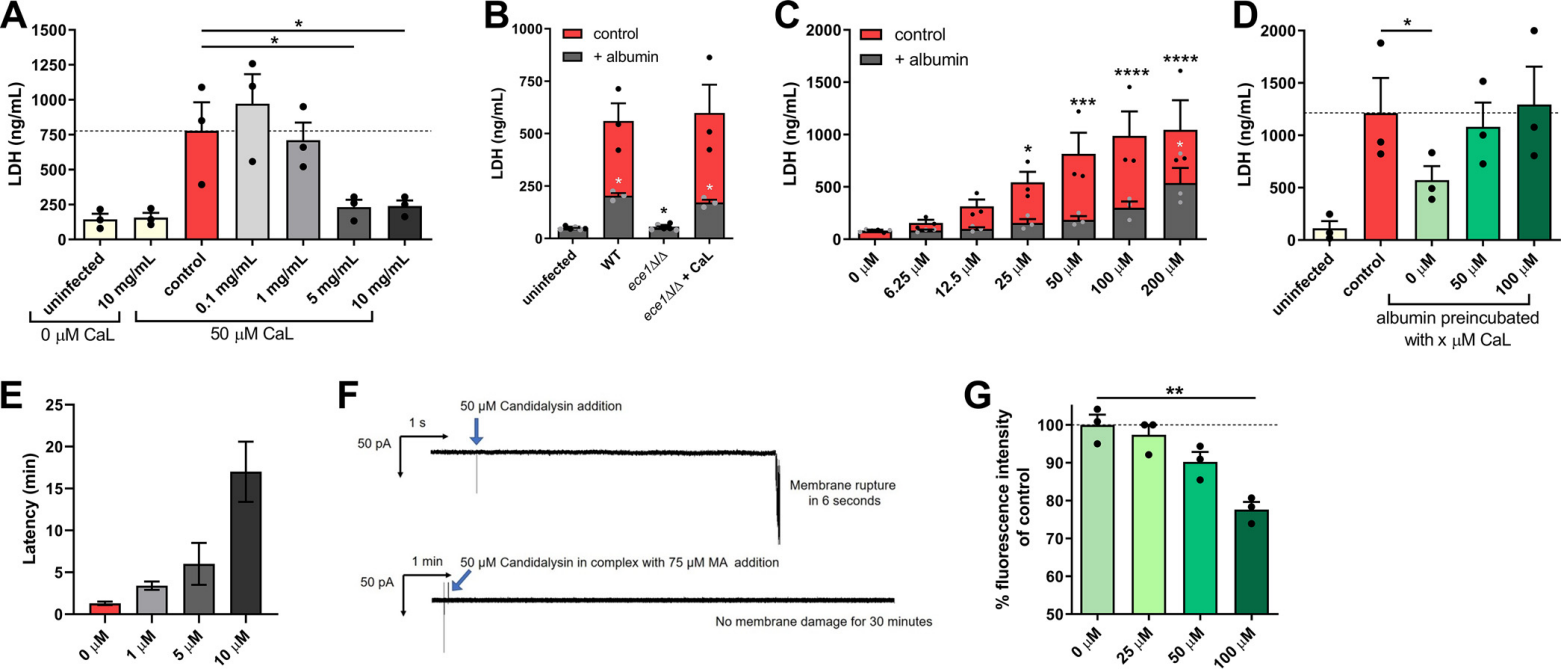
Albumin interferes with candidalysin-induced damage via hydrophobic interactions. (A) Candidalysin (CaL)-induced damage of kidney cells measured by the LDH release 18 h after the addition of 50 μM synthetic candidalysin in the presence of 0 to 10 mg/ml albumin. (B) Damage of kidney cells measured by LDH release after 18 h after infection with the C. albicans wild type (WT) or *ece1*Δ/Δ strains in the presence or absence of 10 mg/ml albumin and the addition of 50 μM candidalysin. (C) Candidalysin-induced damage of kidney cells measured by the LDH release 18 h after the addition of 0 to 200 μM synthetic candidalysin in the presence or absence of 5 mg/ml albumin. (D) Damage of kidney cells measured by LDH release after 18 h in the presence of 5 mg/ml albumin preincubated with 0 to 100 μM synthetic candidalysin and infection with C. albicans. (E) The lipid bilayer membrane damage caused by candidalysin was measured by adding 10 μM candidalysin alone or in a preincubated mixture with different concentrations of mouse albumin (1, 5, and 10 μM) to the lipid bilayer membrane. The latency between the addition of candidalysin and the membrane damage was observed at a constant applied potential of –50 mV in buffer containing 100 mM KCl. Bars represent the mean latency (*n* = 10), and deviations show the SEM. (F) Representative current traces through DPhPC lipid bilayer membrane upon the addition of 50 μM candidalysin alone and in complex with 75 μM mouse albumin. Membrane damage was monitored at a constant potential of –50 mV. 50 μM candidalysin alone caused membrane rupture in 6 s (*n* = 5), while candidalysin in complex with MA did not show any current fluctuations for at least 30 min (*n* = 5). Arrows indicate the addition of candidalysin. (G) Relative fluorescence intensity of ANS bound to human albumin in the presence or absence of 0 to 100 μM candidalysin. Bars represent mean values and the SEM of *n* = 3 independent experiments. For panels A, C, D, and G, a one-way ANOVA with a Dunnett’s posttest correction was used to determine statistical significance compared to the control (= 0 mg/ml albumin or [for panel C] = 0 μM candidalysin) (*, *P* ≤ 0.05; **, *P* ≤ 0.01; ***, *P* ≤ 0.001; ****, *P* ≤ 0.0001). For panel B, significances were calculated using a paired parametric two-tailed Student *t* test (*, *P* ≤ 0.05).

C. albicans-induced host damage relies on the formation of hyphae and secretion of the peptide toxin candidalysin, which is encoded by the *ECE1* gene (30–32). Candidalysin functions by intercalating into host cell membranes, causing cell lysis. Given that albumin prevents C. albicans-induced host damage, we investigated whether albumin interferes with candidalysin activity. Albumin blocked the potential of synthetic candidalysin to damage kidney cells in a dose-dependent manner (Fig. 5A). Similarly, while a candidalysin-deficient *ece1*Δ/Δ strain (nondamaging) supplemented with synthetic candidalysin damaged kidney cells, the addition of albumin prevented damage from occurring (Fig. 5B). This indicates that albumin prevents C. albicans from causing damage by inhibiting candidalysin activity.

To explore its neutralizing capacity, albumin was saturated with increasing concentrations of candidalysin. A 5-mg/ml concentration of albumin completely neutralized the damage capacity of candidalysin up to a concentration of 50 μM (Fig. 5C). Moreover, albumin that was presaturated with candidalysin lost its ability to prevent C. albicans-induced damage (Fig. 5D), suggesting a direct interaction between albumin and candidalysin.

This was confirmed in electrophysiological current measurements, where 10 μM candidalysin (previously optimized) was preincubated with 1, 5, and 10 μM murine albumin and applied to planar lipid bilayers comprising 1,2-diphytanoly-*sn*-glycero-3-phosphocholine (DPhPC). Albumin prevented candidalysin from permeabilizing the lipid bilayer membranes in a dose-dependent manner (Fig. 5E). In addition, under conditions similar to *in vitro* cell damage experiments, preincubation of 50 μM candidalysin with 75 μM murine albumin (equivalent to 5 mg/ml) also abolished membrane permeabilization (Fig. 5F), demonstrating that albumin neutralizes candidalysin cytotoxicity.

Albumin can bind hydrophobic molecules such as fatty acids (33). Notably, candidalysin is an amphipathic peptide with a hydrophobic N terminus and a hydrophilic C terminus (34). To investigate whether albumin interacts with candidalysin via hydrophobic interactions, 8-anilino-1-naphthalenesulfonic acid (ANS) was utilized to analyze the binding of hydrophobic molecules to albumin (35). When bound to hydrophobic sites, ANS can be excited to emit fluorescence, whereas nonbound ANS remains nonfluorescent (35). If hydrophobic molecules occupy the hydrophobic binding sites of albumin, ANS cannot bind and consequently fluorescence intensity decreases depending on direct competition with the other bound molecule (35). Notably, the saturation of albumin with different candidalysin concentrations decreased the fluorescence intensity of ANS in a dose-dependent manner (Fig. 5G). Collectively, these data demonstrate that albumin prevents C. albicans-induced damage by neutralizing candidalysin activity through binding hydrophobic regions of the toxin.

### Candidalysin has no effect in host niches containing albumin.

**FIG 6:**
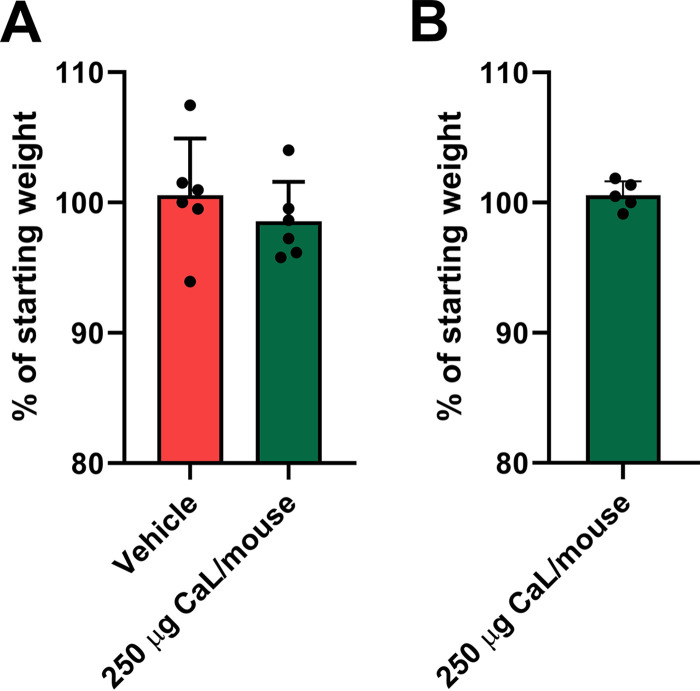
Weight loss after *in vivo* administration of synthetic candidalysin. (A) Percent weight loss compared to starting weights of mice that were intraperitoneally challenged with vehicle or candidalysin (250 μg/mouse) (*n* = 6 mice per group). (B) Percent weight loss compared to starting weights of mice that were intravaginally challenged with candidalysin (250 μg/mouse) (*n* = 5 mice).

**FIG 7:**
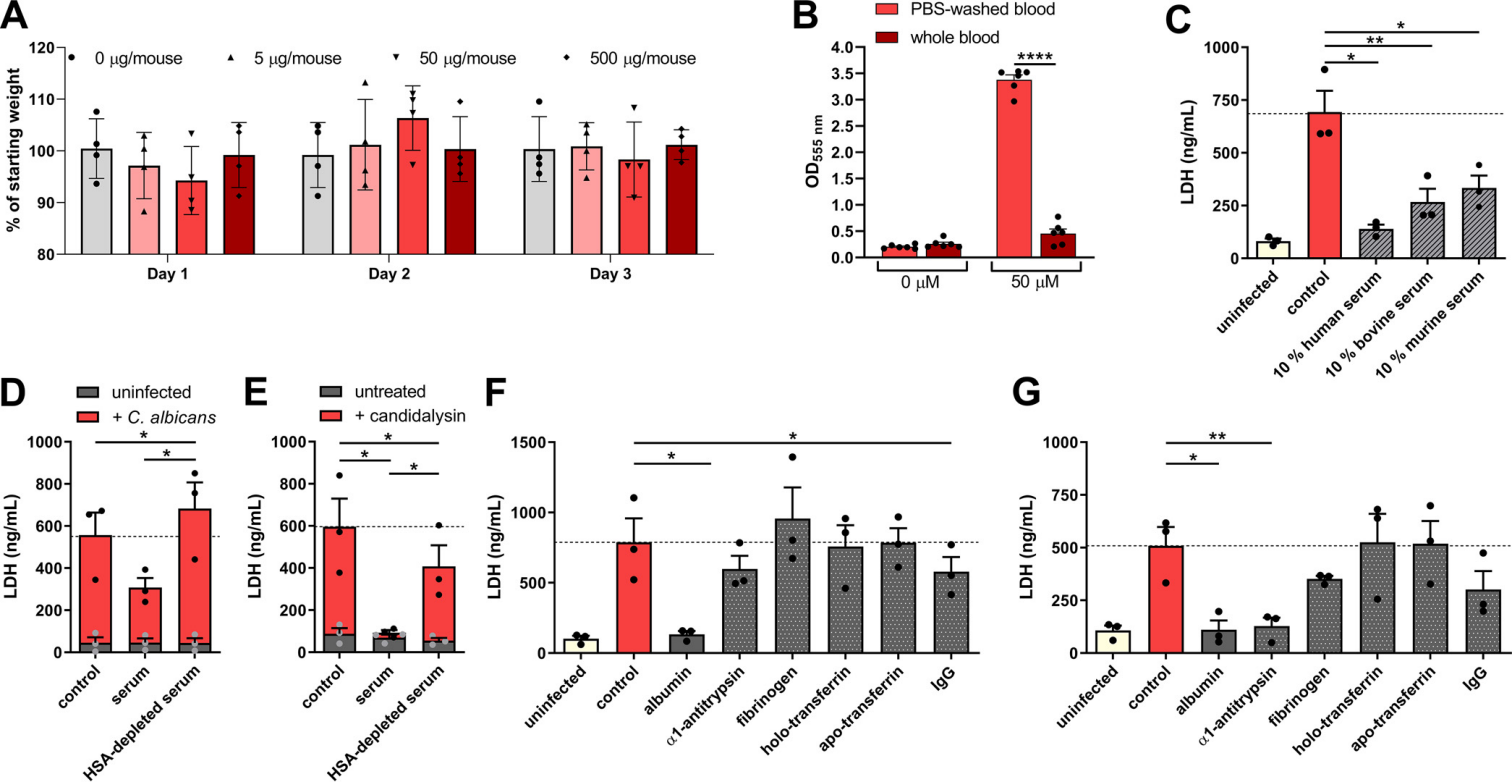
Intravenously administered candidalysin does not elicit disease since it is neutralized by serum. (A) Percent weight loss of starting weight of mice (*n* = 4/group) after intravenous administration via the tail vein with 500, 50, or 5 μg/mouse of candidalysin over 3 days. (B) Absorbance of desoxyhemoglobin measured at 555 nm in whole blood or PBS-washed blood after treatment with 50 μM candidalysin. (C) Damage of kidney cells measured by host cell LDH release 18 h after addition of synthetic candidalysin in the presence of human (HS) serum, 10% bovine (FBS), or murine serum (MS). (D and E) C. albicans (D)- and candidalysin (E)-induced damage to kidney cells in the presence of serum or albumin (HSA)-depleted serum. (F and G) Damage of kidney cells measured by host cell LDH release 18 h after the addition of C. albicans (F) or synthetic 50 μM candidalysin (G) in the presence of abundant serum proteins at their physiological concentrations. Bars represent mean values and the SEM of *n* = 3 (B, D, E, F, and G) or *n* = 6 (C) independent experiments. Significances were calculated using a paired parametric, one-tailed Student *t* test (*, *P* ≤ 0.05; **, *P* ≤ 0.01).

C. albicans SC5314 is a highly pathogenic strain in oral, vaginal, and intravenous models of candidiasis, where pathology is driven by candidalysin (36, 37). Since serum (1), saliva (38), and vaginal fluid (39, 40) contain albumin, we hypothesized that direct administration of candidalysin into the bloodstream, peritoneum, oral cavity or vagina would induce a significantly attenuated host response in comparison to C. albicans infection, due to direct binding and inhibition of candidalysin activity by albumin. Strikingly, no clinical observational effect was noticeable for discomfort, distress, or pain (stary coat, hunching, writhing, abdominal dragging, and lack of locomotion) after (i) intravenous inoculation via the tail vein with 500, 50, or 5 μg of candidalysin/mouse over 3 days; (ii) intraperitoneal administration with 250 μg of candidalysin/mouse over 1 day; (iii) intravaginal administration with 250 μg of candidalysin/mouse over 1 day; or (iv) oral administration directly onto tongue (25 μg of candidalysin/mouse) or sublingually with 2.5, 0.5, or 0.05 μg of candidalysin/mouse over 6 h. In addition, for the oral model, no observable rash or signs of acute inflammation were recorded over the 6 h period. To confirm whether synthetic candidalysin administration had a nonobservational clinical effect (e.g., an influence on appetite), weight loss was recorded in three of the models. No effect on weight loss was observed in the intraperitoneal or intravaginal (at day 1; Fig. 6) or intravenous (over 3 days; Fig. 7A) models. The data strongly support the conclusion that when administered directly, synthetic candidalysin activity may be neutralized by host humoral factors, which likely includes albumin.

### Serum protects against candidalysin-induced damage.

Given that different serum sources can prevent C. albicans induced damage (25) and intravenous candidalysin administration did not elicit clinical effects (Fig. 7A), we confirmed that serum/plasma neutralizes candidalysin. Human blood was centrifuged, and the plasma fraction was removed. When the blood cells were resuspended in phosphate-buffered saline (PBS), the erythrocytes were hemolyzed by candidalysin shown by an increased absorbance of desoxyhemoglobin. In contrast, erythrocytes that were resuspended in plasma were protected against candidalysin-mediated release of hemoglobin (Fig. 7B). Serum (human, bovine, and murine) also neutralizes candidalysin-induced damage in kidney cells (Fig. 7C).

To prove that the damage-neutralizing capacity of serum can be attributed to albumin, we depleted albumin from human serum using affinity column chromatography. Strikingly, albumin-depleted serum lost its capacity to neutralize kidney cell damage induced by C. albicans (Fig. 7D) and candidalysin (Fig. 7E). While albumin is the most abundant serum protein, there are other abundant proteins in serum. When comparing these serum proteins at their physiological concentrations for their capacity to inhibit C. albicans- and candidalysin-induced damage, only albumin was capable of abolishing C. albicans-mediated damage down to uninfected levels (Fig. 7F). However, in addition to albumin, α1-antitrypsin (AAT) reduced candidalysin-induced damage (Fig. 7G). Thus, while albumin may not be the only serum protein capable of neutralizing candidalysin, it appears to be the most efficient one in inhibiting C. albicans-induced cell damage.

### Albumin neutralizes hydrophobic microbial toxins.

Candidalysin is neutralized by albumin via hydrophobic binding interactions. Given this, we hypothesized that other microbial toxins with similar biophysical properties to candidalysin may also be neutralized by albumin.

**FIG 8:**
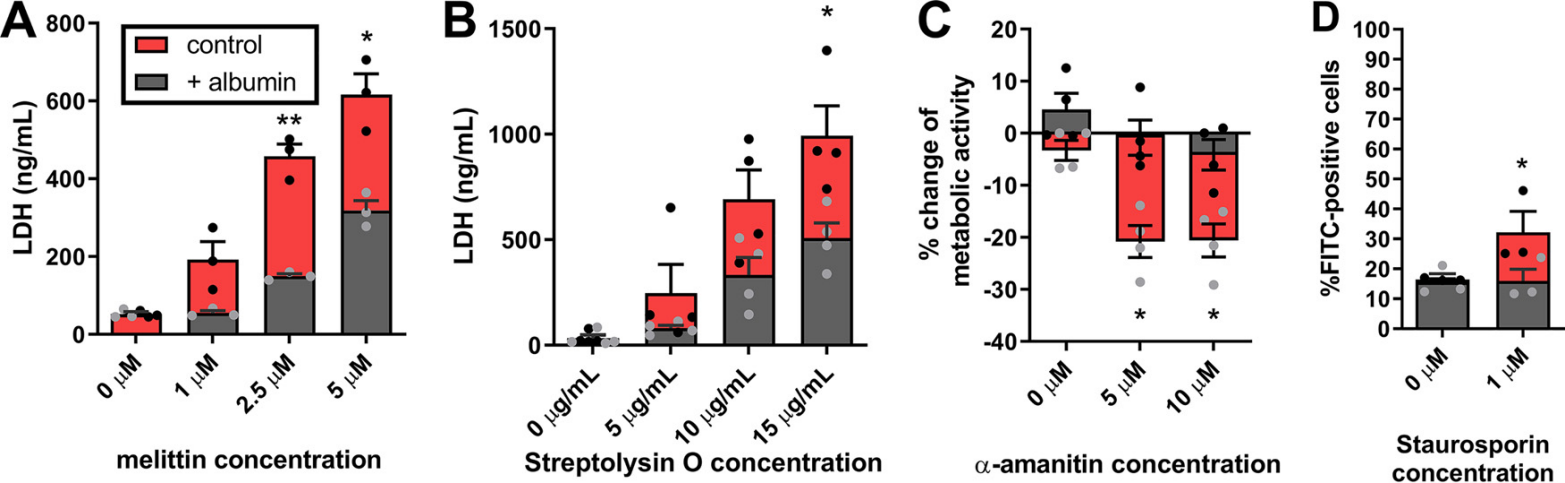
Albumin neutralizes hydrophobic toxins. (A) Damage of kidney cells measured by host cell LDH release 18 h after the addition of 0 to 5 μM the bee venom melittin in the presence or absence of 10 mg/ml albumin. (B) Damage of kidney cells measured by host cell LDH release 18 h after the addition of 0 to 15 μg/ml streptolysin O in the presence or absence of 10 mg/ml albumin. (C) Viability of kidney cells measured by an MTT assay 18 h after exposure to 0 to 10 μM α-amanitin in the presence or absence of 10 mg/ml albumin. (D) Apoptosis of kidney cells measured by Annexin-V staining after 1 h of exposure to 1 μM staurosporin the presence or absence of 5 mg/ml albumin. Bars represent mean values and the SEM of *n* = 3 (A and D) or *n* = 4 (B and C) independent experiments. Significances were calculated using a paired, parametric, two-tailed Student *t* test and compared to the control (= 0 mg/ml albumin) (*, *P* ≤ 0.05; **, *P* ≤ 0.01).

A variety of hydrophobic toxins was selected, which differ in size and mode of action. These included melittin (bee venom; an 26-amino-acid peptide toxin that exhibits a high similarity to candidalysin in terms of structure and amphipathicity [30, 32]), α-amanitin (*Amanita phalloides*; an 8-amino-acid fungal cyclic peptide that inhibits RNA-polymerase II and all translational activity in mammalian cells), staurosporin (*Streptomyces* species; a small molecule bacterial alkaloid that inhibits protein kinases and induces apoptosis), and streptolysin O (Streptococcus pyogenes; a 60-kDa bacterial pore-forming cytolytic protein toxin). Albumin significantly reduced the recognized activities of all four toxins in kidney cells, namely, it reduced cytolytic capacity of melittin and streptolysin O (Fig. 8A and B), recovered metabolic activity of kidney cells after exposure to α-amanitin (Fig. 8C), and reduced the apoptosis-inducing capacity of staurosporin (Fig. 8D). The neutralization of streptolysin O by albumin was also recently demonstrated independent of our findings (41). Interestingly, the predicted binding site of streptolysin O to albumin (41) corresponds to binding site predictions for candidalysin (see Fig. S1 in the supplemental material). In conclusion, albumin exhibits broad protection against a variety of amphipathic/hydrophobic toxins with different modes of action.

10.1128/mBio.00531-21.3 FIG S1 Binding prediction of candidalysin to human albumin. Based on the candidalysin sequence, a PDB file was generated using I-TASSER (81). Three of the predicted candidalysin structures were docked to the structure of human serum albumin (82) using the protein-protein docking tool FRODOCK 2.0 (83). Independent of the predicted candidalysin structure used as input, docking solutions demonstrated a strong tendency to localize to the same region that was predicted to be the binding site of streptolysin O (V310, A306, F374, F377, V381, T341, and L305 [41]). Shown is one of the predicted candidalysin (red) structures docked to human albumin (grey) visualized using Jmol, residues V310, A306, F374, F377, V381, T341, and L305 in human albumin are shown as orange molecules (http://www.jmol.org/). Download FIG S1, TIF file, 2.4 MB. Copyright © 2021 Austermeier et al. 2021 Austermeier et al. https://creativecommons.org/licenses/by/4.0/ This content is distributed under the terms of the Creative Commons Attribution 4.0 International license.

## DISCUSSION

Multiple host factors, including complement proteins and antimicrobial peptides, are known to antagonize microbial pathogens. However, the influence of conventional host molecules such as abundant serum proteins on host-pathogen interactions during infection remains poorly explored. Here, we elucidated the influence of albumin, one of the most common human proteins, on the pathogenicity of the model fungal pathogen C. albicans. This yeast was chosen for its unique ability among microbial pathogens to infect nearly every tissue and organ of the human body. Our study demonstrates that albumin prevented C. albicans-induced damage in intestinal, vaginal, endothelial, liver, and kidney cells and reduced the ability of C. albicans to translocate across intestinal epithelial cells. Albumin functions by inhibiting the cytolytic peptide toxin candidalysin by interacting with its hydrophobic region. Importantly, albumin also neutralizes a wide range of hydrophobic bacterial and fungal toxins, demonstrating a role for albumin in host defense against toxins.

Previous studies have demonstrated conflicting findings regarding C. albicans-albumin interactions. While one study suggested that physiological concentrations of albumin (5 to 50 mg/ml) had inhibitory and even antimycotic effects on C. albicans (42), another study indicated a growth-promoting effect through the utilization of albumin-bound heme as an iron source (29, 43). Our study confirms the latter observation as albumin restored the ability of C. albicans to proliferate in the presence of the iron-chelating agent BPS. Given that C. albicans can utilize bovine albumin as a source of nitrogen via proteolytic degradation using its secreted aspartic proteases (44–46), human albumin also likely acts as a nitrogen source for C. albicans. Consequently, the growth-promoting effect of albumin on C. albicans on IECs most likely reflects two nutritional properties: the utilization of albumin as both an iron and a nitrogen source. In addition, we observed that preincubation with albumin is sufficient to promote C. albicans growth. However, this growth-promoting effect is not as strong compared to C. albicans infection in the constant presence of albumin.

The protective effect of albumin against C. albicans- and candidalysin-induced damage in all cell types tested has significant implications for fungal mucosal and systemic infections. Since preincubation of host or fungal cells failed to reduce damage, this suggests that albumin acts by neutralizing candidalysin during its release by invading C. albicans hyphae during infection. Electrophysiological current measurements of lipid bilayer membranes confirmed that albumin interacts with candidalysin preventing membrane binding. Furthermore, using the fluorescent marker molecule ANS, albumin (net-negative charge) inhibition of candidalysin (net-positive charged) activity appears to be mediated through hydrophobic interactions between the molecules rather than electrostatic interactions. Notably, α1-antitrypsin (AAT), which harbors a conserved hydrophobic surface pocket (47) and can bind fatty acids similar to albumin (48, 49), was also able to neutralize candidalysin. However, since AAT only protected against candidalysin-mediated damage but not C. albicans-induced damage, this suggests that albumin is either more inhibitory than AAT or has additional effects on the fungus in addition to neutralizing candidalysin activity. Interestingly, we observed more prominent protective effects of albumin on organ cell lines compared to the epithelial cell lines. Epithelial cells efficiently endocytose albumin (50), which may have reduced the amount of albumin that could interact with candidalysin on epithelial cells compared to organ cells, resulting in a stronger protective effect on cells that are not endocytosing albumin.

Our data suggest that albumin is a key player in the physiology of C. albicans infections and disease pathogenesis. This is further supported by our observation that neither intravenous, nor intraperitoneal, nor intravaginal, nor oral administration of synthetic candidalysin led to (observational) clinical effects commonly observed during systemic candidiasis in mice (51). This is most likely due to the abundant presence of albumin at these body sites that may efficiently neutralize candidalysin activity. These data are highly intriguing considering the critical role of candidalysin during mucosal and systemic C. albicans infections (36, 37). Given that candidalysin is thought to be secreted by invading hyphae only in the invasion pocket, we propose either that albumin cannot enter the invasion pocket in sufficient quantities to neutralize candidalysin activity or candidalysin rapidly intercalates into host cell membranes as soon as it is secreted (thus, not providing sufficient time for albumin to neutralize it). These hypotheses are supported by our observation that the addition of albumin after the initiation of hyphal invasion resulted in a diminished protective effect.

Historically, albumin has been reported to have both beneficial and detrimental functions during microbial infections. For example, with regard to pathogenic fungi, albumin exhibits a growth-inhibitory effect on Blastomyces dermatitidis (52), can disrupt extracellular vesicles produced by Cryptococcus neoformans (53), and acts synergistically with antimycotic agents against Aspergillus hyphae (54) but conversely increases the MICs of antifungals against A. fumigatus (55). With regard to pathogenic bacteria, albumin facilitates survival of group G streptococci on epithelial surfaces (56), can impact Acinetobacter baumannii survival and persistence (57, 58), and binds homoserine lactones, thereby interfering with P. aeruginosa quorum sensing (59). Our study now provides evidence that albumin is also a critical player during *Candida albicans* infections.

A key finding was our discovery that albumin can neutralize candidalysin activity. This is the first demonstration of albumin neutralizing a fungal (peptide) toxin. Since toxins are highly variable in their structure and function, we confirmed the neutralizing effect of albumin against several toxins, including a cytolytic peptide (melittin) and two noncytolytic toxins (α-amanitin and staurosporin). This demonstrates that the neutralizing effect of albumin is independent of a toxin’s mode of action but rather relates to their common biophysical hydrophobic properties. Interestingly, albumin also has been observed to neutralize Clostridioides difficile TcdA and TcdB toxins, which was predicted to be mediated through hydrophobic interactions (60). Similarly, the Streptococcus pyogenes toxin streptolysin O is neutralized by albumin (41), which we also observed. Interestingly, *in silico* binding predictions suggest the same binding region for C. difficile toxins (60), streptolysin O (41), and candidalysin. Therefore, we conclude that during microbial infection albumin functions as a neutralizing protein of hydrophobic toxins.

The clinical relevance of albumin in disease is clear. Reduced circulating albumin concentrations correlate with an enhanced all-cause mortality rate (61) and all-cause mortality in hospitalized patients (62, 63). Also, under acute inflammatory conditions, albumin production in the liver is reduced (64–66), with lower albumin levels correlating with the onset of infections and increased mortality in critically ill patients (67, 68). Low albumin levels are also a recognized risk factor for developing pneumonia with Pneumocystis jirovecii (69) and as a mortality-associated risk factor in candidemia (70) or fungal peritonitis (71) patients. In contrast, increased albumin levels have been correlated with reduced susceptibility to bacteremia in burn patients (72) and used as a marker of infection in the vaginal mucus of pregnant women (73). Furthermore, increased albumin levels correlated with increased survival of patients with C. difficile infection, as well as *Candida* spp., *Enterobacteriaceae*, or Klebsiella pneumoniae-caused bloodstream infections (74). Although these studies suggest a role for albumin in infections, adult patients suffering from the rare disease congenital analbuminemia do not suffer from a severely heightened susceptibility to infectious diseases (75). This underlines that the combination of low albumin levels with underlying disease or confounder effects might have driven the associations between reduced albumin levels and infectious diseases. Therefore, the role of albumin during candidiasis requires further in-depth clinical and mechanistic elucidation to identify whether it can serve as a biomarker or can be used for therapy.

In summary, albumin is widely known to be a binding partner of endogenous and exogenous molecules, but it is becoming increasingly apparent that albumin also plays a role in the detoxification of hydrophobic microbial toxins. In C. albicans infections, albumin promotes fungal proliferation by acting as a nutrient but simultaneously diminishing the ability of the fungus to damage the host by neutralizing its peptide toxin candidalysin. As such, our work identifies a novel mechanism by which albumin can modulate infection, potentially identifying albumin as an important player in defense against microbial toxins.

## MATERIALS AND METHODS

### *C. albicans* strains.

Strain SC5314 (76) was used as the C. albicans wild-type strain. For experiments with the *ece1*Δ/Δ strain (30), the parental strain BWP17 CIp30 (77) was used as a wild-type control. Strains from glycerol stocks were streaked out and maintained on yeast extract-peptone-dextrose (YPD) plates for a maximum of 2 weeks. For experiments, 20 ml of YPD broth was inoculated with a single colony of C. albicans, followed by incubation overnight at 30°C and 180-rpm orbital shaking. The next day, yeasts were harvested by centrifugation, washed three times using PBS (pH 7.4), enumerated using a Neubauer chamber, and adjusted to the concentration required for the subsequent experiment.

### Serum and human blood experiments.

Human serum was obtained from venous blood samples donated from healthy volunteers that gave written informed consent. The Jena institutional ethics committee approved the procedure (Ethik-Kommission des Universitätsklinikums Jena, permission 2207-01/08).

For experiments with whole blood, blood was transferred into Eppendorf tubes and centrifuged at 3,000 × *g* for 5 min. The plasma fraction was either used for resuspension or removed, and cells were resuspended in PBS (pH 7.4). After repeating the washing step three times, 50 μM candidalysin was added, and cells were incubated at 37°C and 5% CO_2_. After 1 h, the samples were centrifuged again, and the supernatant was transferred to a 96-well plate. The absorbance of desoxyhemoglobin was measured at 555 nm in a Tecan M-Plex reader.

For serum preparation, blood was collected in 5.5-ml S-Monovette tubes (Sarstedt), allowed to coagulate for at least 30 min, and centrifuged for 10 min at 2,000 × *g*. The serum was collected and stored at −20°C until use.

Depletion of albumin from serum was done according to the protocol supplied by the manufacturer (Abcam). In brief, albumin binding spin columns were equilibrated with albumin binding buffer. Serum was diluted 1:10, transferred to the column, and incubated for 30 min at 400-rpm orbital shaking and room temperature. After incubation, the columns were centrifuged for 1 min at 200 × *g*, and the flowthrough containing the albumin-depleted serum fraction was collected and used for experiments with albumin-depleted serum.

Fetal bovine serum (FBS) was bought commercially (Bio & Sell), and murine serum was obtained from C57BL/6J and CD-1 mice.

### Chemicals and proteins.

In all experiments, human albumin from Sanquin Plasma Products B.V. (Albuman) was used unless otherwise indicated. In selected experiments human albumin from other suppliers was used (Bio & Sell catalog number HSA.FV.0025 and Sigma catalog number A6909), bovine serum albumin (BSA; Bio & Sell Fraction V catalog number BS.BSA.FV0100), murine serum albumin (MA; Abcam, catalog number ab183228), albumin crude from chicken egg (ovalbumin; Pan Reac AppliChem catalog number A4344,0250). To exclude small-molecule preservatives and contaminations, albumin from Sanquin Plasma Products B.V. (Albuman) was filtered based on the molecular weight using 30-kDa MWCO (molecular weight cutoff) filters (Pall Corporation). The column was equilibrated by adding double-distilled H_2_O (H_2_O_dd_) and centrifugation for 5 min at 4,000 × *g*. Albumin solution was diluted 1:2 in H_2_O_dd_ and centrifuged over the column for 40 min at 4,000 × *g*.

Other protein preparations include human apo-transferrin (Sigma), human holo-transferrin (Calbiochem, Merck), human fibrinogen (Sigma), human α1-antitrypsin (Zemaira; CSL Behring), and human immunoglobulin G (Nanogam; Sanquin Plasma Products B.V.). Chemicals used include bathophenanthrolinedisulfonic acids disodium salt (BPS; Alfa Aesaer), 8-anilino-1-naphthalensulfonic acid (ANS; Sigma), glycylglycine (Alpha Aesar), and thiazolyl blue (MTT; Sigma). Toxins used include synthetic candidalysin (Peptide Synthetics), melittin (Proteogenix), α-amanitin from *Amanita phalloides* (Sigma-Aldrich), streptolysin O from Streptococcus pyogenes (Sigma-Aldrich), and staurosporin (Abcam).

### Culture and maintenance of human intestinal epithelial cells.

The intestinal epithelial cell line C2BBe1 (Caco-2 brush border expressing 1; ATCC, CRL2102) and human intestinal goblet cell HT29-MTX (ATCC, HBT-38; CLS lot 13B021) were handled as previously described (28). In brief, cells were maintained at 37°C and 5% CO_2_ in Dulbecco modified Eagle medium (DMEM; Gibco, Thermo Fisher Scientific) containing 10% heat-inactivated FBS (Bio & Sell), 10 μg/ml holo-transferrin (Calbiochem, Merck), and 1% nonessential amino acids (Gibco, Thermo Fisher Scientific). Cell lines have been authenticated *via* commercial STR profiling (Eurofins Genomic) and checked for contaminations using a PCR mycoplasma test kit (PromoKine) according to the protocol supplied by the manufacturer. For cell seeding, transwell inserts comprising a polycarbonate membrane with 5-μm pores (Corning) or the wells of a 96-well plate were coated with 10 μg/ml collagen I overnight at 4°C (Thermo Fisher Scientific). The next day, coated transwell inserts or wells were washed twice using H_2_O_dd_, and the C2BBe1:HT29-MTX cell mixture was seeded at a ratio of 70:30 and a density of 2 × 10^4^ cells/well. Transwell inserts were placed in 24-well plates, and the lower compartment was filled with supplemented DMEM as well. For differentiation, intestinal epithelial cells were cultured for 14 days under humidified conditions, and the medium was exchanged after 7 days. Below, the C2BBe1:HT29-MTX cell mixture will be referred to as IECs.

### Culture and maintenance of human vaginal cells.

Human vaginal epithelial cells A-431 (DSMZ no. ACC 91) and primary human vaginal cells (ATCC PCS-480-010) were handled according to the suppliers’ instructions. A-431 cells were cultivated in RPMI 1640 medium (Thermo Fisher Scientific) supplemented with 10% FBS (Bio & Sell). Primary vaginal cells were cultured in vaginal epithelial cell basal medium (ATCC PCS-480-030), supplemented with components from a vaginal epithelial cell growth kit (ATCC PCS-480-040). Both cell lines were seeded at a density of 4 × 10^4^ cells/well in a 96-well plate, followed by incubation in a humidified incubator at 37°C and 5% CO_2_ until confluence was reached. The A-431 cell line has been authenticated via commercial STR profiling (Eurofins Genomic) and checked for contaminations using a PCR mycoplasma test kit (PromoKine) according to the manufacturer’s instructions.

### Culture and maintenance of human endothelial cells.

Human umbilical cord vein endothelial cells (HUVECs) were isolated, expanded in 150-cm^2^ flasks in endothelial cell medium (ECM; Promocell), and frozen in liquid nitrogen in medium (1 × 10^6^/ml) containing 9% FBS and 7.5% dimethyl sulfoxide (DMSO). For experiments, endothelial cells from glycerol stocks were grown in 150-cm^2^ flasks for 72 h, harvested, and seeded at a concentration of 2 × 10^4^ cells/well in a 96-well plate for damage assays. HUVECs were incubated at 37°C and 5% CO_2_ until they reached confluence.

### Culture and maintenance of human liver cell.

The hepatocyte cell line HepaRG was handled according to the supplier instructions (Invitrogen). Hepatocytes were cultivated at 37°C and 5% CO_2_ in William’s E medium (Gibco, Thermo Fisher Scientific) supplemented with HepaRG Thaw, Plate, & General Purpose Medium Supplement (Gibco, Thermo Fisher Scientific). HepaRG were seeded at a density of 4 × 10^4^ cells/well in a 96-well plate and grown at 37°C and 5% CO_2_ until they reached confluence.

### Culture and maintenance of human kidney cells.

The kidney cell HEK293 subclone HEK293A (human embryonic kidney cells transformed with sheared human adenovirus type 5 DNA) was handled according to the supplier instructions (Invitrogen). In brief, cells were maintained at 37°C and 5% CO_2_ in DMEM containing 10% heat-inactivated FBS. HEK293A cells were seeded at concentrations of 3.5 × 10^5^ cells/well in a 6-well plate for flow cytometry analysis, 1 × 10^5^ cells/well in a 24-well plate for PI staining, or 2 × 10^4^ cells/well in a 96-well plate for damage assays and grown at 37°C and 5% CO_2_ until they reached confluence. Cell lines have been authenticated via commercial STR profiling (Eurofins Genomic) and checked for contaminations using a PCR mycoplasma test kit (PromoKine) according to the manufacturer’s instructions.

### Translocation assays with intestinal epithelial cells.

Translocation assays were performed on IECs in transwell inserts as described previously (27) with minor modifications. Experiments were done in technical triplicates for infected samples and in technical duplicates for uninfected samples. Before infection, medium in the lower compartment was exchanged to DMEM with or without 5 mg/ml human albumin (Albuman; Sanquin Plasma Products B.V.) and incubated for 2 h at 37°C and 5% CO_2_. After 2 h, cells were infected with 2 × 10^4^ C. albicans cells/transwell in DMEM and further incubated at 37°C and 5% CO_2_. For quantification of adhesion, the supernatant of transwells was removed after 1 h of infection. Afterward, the transwells were once washed with PBS and added to the collected supernatant (= nonadherent C. albicans). The remaining cells of transwells (= adherent) were resuspended in PBS and collected separately. To determine the CFU, collected samples were plated on YPD plates. Alternatively, after 18 h of infection, the transepithelial electrical resistance (TEER) was measured with a volt-ohm meter (WPI). Zymolyase (20 U/ml; Amsbio) was then added to the lower compartment, followed by incubation at 37°C and 5% CO_2_. After 2 h, the zymolyase-treated C. albicans cells in the lower compartment, as well as the C. albicans cells grown on the C2BBe1 cells, were collected separately and plated on YPD agar for CFU quantification.

### Growth curve analysis with *C. albicans*.

A total of 2 × 10^4^ C. albicans cells/well were grown in the presence or absence of 10 mg/ml human albumin (Albuman; Sanquin Plasma Products B.V.) and 1 mM BPS in RPMI 1640 (Gibco, Thermo Fisher Scientific) in a 96-well plate sealed with foil and incubated at 37°C in a Tecan M-Plex reader. The OD_600_ was measured every 30 min. Before each measurement, the plate was shaken for 15 s at 140 rpm. During the measurement, nine positions per well were measured with five flashes.

### Live-cell imaging of cell death dynamics using propidium iodide.

Live-cell imaging of the cell death dynamics using PI staining was performed as described previously (78). In brief, a confluent monolayer of HEK293A cells in a 24-well plate was washed once with culture medium and subsequently infected with 1 × 10^5^ C. albicans yeast cells in culture medium or culture medium containing human albumin (Albuman; Sanquin Plasma Products B.V.) concentrations between 10 mg/ml and 0.1 mg/ml. All media contained 4 μg/ml propidium iodide (Sigma-Aldrich). Cells were imaged in a Zeiss Celldiscoverer 7 for 18 h at 37°C and 5% CO_2_. Microscopy pictures were taken every 20 min at 10 × magnification from four independent positions per well in the bright field, as well as fluorescence with excitation at 353 nm, and emission was recorded at a wavelength of 465 nm.

Microscopy pictures from the red fluorescence channel were analyzed using Fiji (79). Using the threshold function, images were converted to binary images, and the number of PI-positive nuclei was enumerated for each image using macro batch analysis and the Particle Analyzer tool. Based on the obtained counts of PI-positive kidney cells, the percentage of dead kidney cells was calculated in relation to the maximal number of dead cells.

### Lactate dehydrogenase cell damage assays.

Before infection, cell lines (IECs, HEK293A, A-431) or primary cells (HUVECs) were washed once in their respective medium without FBS. Then, respective medium or medium containing human albumin (0.1 to 35 mg/ml), bovine albumin (10 mg/ml), murine albumin (10 mg/ml), ovalbumin (10 mg/ml), AAT (1.5 mg/ml), fibrinogen (2 mg/ml), holo-transferrin (3 mg/ml for physiological concentration, otherwise 10 mg/ml), apo-transferrin (3 mg/ml for physiological concentration, otherwise 10 mg/ml), IgG (10 mg/ml), or 10% serum was added, respectively. This was immediately followed by the addition of the 2 × 10^4^/well C. albicans, synthetic candidalysin (6.25 to 200 μM), melittin (1 to 5 μM), or streptolysin O (5 to 15 μg/ml). Cells were incubated for 18 h at 37°C and 5% CO_2_. After 18 h, the plate was centrifuged at 250 × *g* for 10 min. Supernatants were taken, diluted, and analyzed according to the manufacturer’s instructions of the cytotoxicity kit (Roche). LDH release was quantified based on a standard curve prepared from a 5-mg/ml stock obtained from rabbit muscle (Roche).

For preincubation of HEK293A cells with albumin previous to infection, DMEM containing 10 mg/ml human albumin (Albuman; Sanquin Plasma Products B.V.) was added, and HEK293A cells were incubated for 1 h at 37°C and 5% CO_2_. After 1 h, HEK293A cells were washed once with DMEM and infected with 2 × 10^4^ C. albicans cells/well in DMEM. Alternatively, for preincubating C. albicans cells with albumin, 1 × 10^5^ C. albicans cells/ml were incubated for 1 h at 37°C and 400-rpm orbital shaking in DMEM plus 10 mg/ml human albumin (Albuman; Sanquin Plasma Products B.V.). After 1 h, cells were centrifuged for 3 min at 4,000 × *g*, resuspended in the same volume, and HEK293A cells were infected with 2 × 10^4^ C. albicans cells/well. The experiment was then continued with the 18 h of incubation as described above. For CFU plating of grown C. albicans on the cell layer, the content of the well was scraped, transferred to a 1.5-ml Eppendorf tube, diluted in PBS, and plated on YPD agar plates. Colonies were counted after incubation for 2 days at 30°C.

The effects of delayed albumin addition were tested by adding 10 mg/ml albumin (Albuman; Sanquin Plasma Products B.V.) after 0, 4, and 6 h, respectively. In addition, all conditions during the infection were kept the same as described above.

For saturation experiments with albumin, human albumin (Albuman; Sanquin Plasma Products B.V.) was preincubated with a respective concentration of candidalysin (0 to 100 μM) for 1 h at 37°C and 400-rpm orbital shaking. Afterward, the candidalysin-preexposed albumin was added to HEK293A cells, and cells were infected with 2 × 10^4^ C. albicans cells/well. Supernatants for LDH determination were taken after 18 h, and measurements were made as described earlier.

### Murine candidalysin treatment models.

Female BALB/c or C57BL/6 mice (22 to 25 g) were used for all experiments. For intravenous administration, mice were inoculated via the tail vein at 500, 50, or 5 μg/mouse with candidalysin (in 100 μl of sterile saline) (*n* = 4 per group). For vaginal and intraperitoneal administration, candidalysin at 250 μg/mouse (in 100 μl of sterile saline) was administered intravaginally or intraperitoneally, respectively (*n* = 5/6 per group). For oral administration, mice were first anesthetized with 75 mg/kg ketamine and 1 mg/kg medetomidine (Domitor) by intraperitoneal injection (100 μl/10 g). Once anesthetized, candidalysin at 25 μg/mouse (in 100 μl of sterile saline) was applied directly onto the tongue (*n* = 5 per group) or candidalysin at 2.5, 0.5, or 0.05 μg/mouse (in 10 μl of sterile saline) was applied sublingually (*n* = 2 per group). Animals were kept with their heads placed in anteflexion for 30 min (sublingually) or 60 min (tongue). After the procedure, mice received 1 mg/kg medetomidine antagonist atipamezole (Antisedan) by intraperitoneal injection and were allowed to recover from anesthesia in a heated cage. All models were assessed for clinical phenotypes of discomfort, distress, or pain, including stary coat, hunching, writhing, abdominal dragging, and lack of locomotion (up to 6 h for oral, up to 24 h [day 1] for intravaginal and intraperitoneal, and for 3 days for intravenous). For the oral model, the presence of a rash or any sign of acute inflammation was also recorded for the 6-h period. Mice were weighed prior to termination on day 1 for the intravaginal and intraperitoneal models and over 3 days for the intravenous model.

### MTT assay.

MTT assay for the quantification of the metabolic activity of cells exposed to α-amanitin was done as described previously (80) with minor modifications. In brief, HEK293A cells were exposed to 0 to 10 μM α-amanitin in the presence or absence of 10 mg/ml human albumin (Albuman; Sanquin Plasma Products B.V.) at 37°C and 5% CO_2_. After 18 h, the medium was replaced by DMEM containing 0.5 mg/ml MTT and incubated for 1 h at 37°C and 5% CO_2_. Afterward, an equal amount of DMSO was added to dissolve the formazan crystals, and samples were incubated for a further 30 min. The absorption of samples was measured at 570 nm in a Tecan M-Plex reader.

### Annexin-V staining.

HEK293A cells were exposed to 1 μM staurosporin in the presence or absence of 5 mg/ml human albumin (Albuman; Sanquin Plasma Products B.V.). After 1 h of incubation at 37°C and 5% CO_2_, cells were stained for Annexin-V expression according to the manufacturer’s instructions (Abcam) with minor modifications. In brief, cells were washed once with assay buffer; assay buffer containing Apopxin green indicator for staining of apoptotic cells was then added, followed by incubation at room temperature. After 30 min, the staining solution was removed, the cells were washed once with assay buffer, and the percentage of Annexin-V-positive cells was measured on a FACSVerse (BD Bioscience) flow cytometer. Data analysis was performed using FlowJo version 10.6.1.

### ANS binding assay for quantification of candidalysin binding to albumin.

The binding assay of candidalysin to hydrophobic pockets of human albumin was based on 8-anilino-1-naphthalenesulfonic acid (ANS) competition assay described previously (35). First, 1 μmol fatty acid-free albumin (Bio & Sell) was preincubated in 0.1 mol/liter glycylglycine buffer (pH 7.4) with 0 to 100 μM candidalysin at 37°C. After 1 h, 10 μmol of ANS was added, and samples were incubated for 20 min at room temperature. The fluorescence intensity of ANS was measured in a Tecan M-Plex reader at an excitation wavelength of 373 nm, and the emission was recorded at 474 nm.

### Electrical current recording and data analysis.

Electrophysiological current measurements were performed using parallel planar lipid bilayers with Orbit 16 (Nanion). The horizontal bilayers were formed over 16-channel multielectrode-cavity-array (MECA) chips (Ionera) using 1,2-diphytanoly-*sn*-glycero-3-phosphocholine (DPhPC; Avanti Polar Lipids) dissolved in octane (25 mg ml^−1^). Both *cis* (grounded) and *trans* cavities above and below the bilayers were filled with electrolyte solution (150 μl total) containing 0.1 M KCl and 20 mM HEPES (pH 7.4). Reconstituted candidalysin peptides in water were added to the *cis* side of the bilayers to obtain a final concentration of 10 μM. For the complex formation with murine albumin (MA; Abcam), 100 μM candidalysin were incubated with 10, 50, and 100 μM MA, respectively, in a 15-μl volume at room temperature for 10 min. After 15 μl of buffer was removed from the *cis* side of the bilayers, 15 μl of the preincubated candidalysin-MA mix was added to the same side and mixed well by pipetting, yielding a final concentration of 10 μM candidalysin with 1, 5, and 10 μM MA, respectively. A constant voltage of –50 mV was applied, and current changes were monitored at room temperature. Current traces were acquired at a sampling frequency of 10 kHz using Element Data Recorder software (EDR 3.8.3). Current analysis was performed using Clampfit (Molecular Devices).

### Statistical analysis.

Experiments were usually done in biological triplicates (*n* = 3) unless indicated differently. Graphs show the mean values of biological replicates, including the standard errors of the mean (SEM). GraphPad Prism 8 was used for analyses. In order to test for significance, either two-tailed or one-tailed paired and parametric *t* tests or one-way analysis of variance (ANOVA), including a Dunnett’s posttest, were done. Statistical significances always refer to the control (= 0 mg/ml albumin) and are indicated by asterisks in the figures (*, *P* ≤ 0.05; **, *P* ≤ 0.01; ***, *P* ≤ 0.001; ****, *P* ≤ 0.0001).

## References

[1] Merlot AM, Kalinowski DS, Richardson DR. 2014. Unraveling the mysteries of serum albumin-more than just a serum protein. Front Physiol 5:299. doi:10.3389/fphys.2014.00299. 25161624 PMC4129365

[2] van der Vusse GJ. 2009. Albumin as fatty acid transporter. Drug Metab Pharmacokinet 24:300–307. doi:10.2133/dmpk.24.300. 19745557

[3] Taverna M, Marie AL, Mira JP, Guidet B. 2013. Specific antioxidant properties of human serum albumin. Ann Intensive Care 3:4. doi:10.1186/2110-5820-3-4. 23414610 PMC3577569

[4] Levitt DG, Levitt MD. 2016. Human serum albumin homeostasis: a new look at the roles of synthesis, catabolism, renal and gastrointestinal excretion, and the clinical value of serum albumin measurements. Int J Gen Med 9:229–255. doi:10.2147/IJGM.S102819. 27486341 PMC4956071

[5] Dvorak HF. 2010. Vascular permeability to plasma, plasma proteins, and cells: an update. Curr Opin Hematol 17:225–229. doi:10.1097/MOH.0b013e3283386638. 20375889 PMC2878124

[6] Drell T, Lillsaar T, Tummeleht L, Simm J, Aaspollu A, Vain E, Saarma I, Salumets A, Donders GG, Metsis M. 2013. Characterization of the vaginal micro- and mycobiome in asymptomatic reproductive-age Estonian women. PLoS One 8:e54379. doi:10.1371/journal.pone.0054379. 23372716 PMC3553157

[7] Pierce JV, Kumamoto CA. 2012. Variation in *Candida albicans* EFG1 expression enables host-dependent changes in colonizing fungal populations. mBio 3:e00117-12. doi:10.1128/mBio.00117-12. 22829676 PMC3413400

[8] Witchley JN, Penumetcha P, Abon NV, Woolford CA, Mitchell AP, Noble SM. 2019. *Candida albicans* morphogenesis programs control the balance between gut commensalism and invasive infection. Cell Host Microbe 25:432–443 e6. doi:10.1016/j.chom.2019.02.008. 30870623 PMC6581065

[9] Montelongo-Jauregui D, Lopez-Ribot JL. 2018. *Candida* interactions with the oral bacterial microbiota. J Fungi (Basel) 4:122. doi:10.3390/jof4040122. PMC6308928 30400279

[10] Mayer FL, Wilson D, Hube B. 2013. *Candida albicans* pathogenicity mechanisms. Virulence 4:119–128. doi:10.4161/viru.22913. 23302789 PMC3654610

[11] Cheng MF, Yang YL, Yao TJ, Lin CY, Liu JS, Tang RB, Yu KW, Fan YH, Hsieh KS, Ho M, Lo HJ. 2005. Risk factors for fatal candidemia caused by *Candida albicans* and non-albicans *Candida* species. BMC Infect Dis 5:22. doi:10.1186/1471-2334-5-22. 15813977 PMC1090575

[12] Koh AY, Kohler JR, Coggshall KT, Van Rooijen N, Pier GB. 2008. Mucosal damage and neutropenia are required for *Candida albicans* dissemination. PLoS Pathog 4:e35. doi:10.1371/journal.ppat.0040035. 18282097 PMC2242836

[13] Zhai B, Ola M, Rolling T, Tosini NL, Joshowitz S, Littmann ER, Amoretti LA, Fontana E, Wright RJ, Miranda E, Veelken CA, Morjaria SM, Peled JU, van den Brink MRM, Babady NE, Butler G, Taur Y, Hohl TM. 2020. High-resolution mycobiota analysis reveals dynamic intestinal translocation preceding invasive candidiasis. Nat Med 26:59–64. doi:10.1038/s41591-019-0709-7. 31907459 PMC7005909

[14] Lionakis MS, Lim JK, Lee CC, Murphy PM. 2011. Organ-specific innate immune responses in a mouse model of invasive candidiasis. J Innate Immun 3:180–199. doi:10.1159/000321157. 21063074 PMC3072204

[15] Kullberg BJ, Arendrup MC. 2015. Invasive candidiasis. N Engl J Med 373:1445–1456. doi:10.1056/NEJMra1315399. 26444731

[16] Kumamoto CA, Gresnigt MS, Hube B. 2020. The gut, the bad, and the harmless: *Candida albicans* as a commensal and opportunistic pathogen in the intestine. Curr Opin Microbiol 56:7–15. doi:10.1016/j.mib.2020.05.006. 32604030 PMC7744392

[17] Martinez JP, Lopez-Ribot JL, Chaffin WL. 1994. Heterogeneous surface distribution of the fibrinogen-binding protein on *Candida albicans*. Infect Immun 62:709–712. doi:10.1128/iai.62.2.709-712.1994. 8300229 PMC186163

[18] Schultz CM, Goel A, Dunn A, Knauss H, Huss C, Launder D, Wuescher LM, Conti HR, Worth RG. 2020. Stepping up to the plate(let) against *Candida albicans*. Infect Immun 88:e00784-19. doi:10.1128/IAI.00784-19. 31932331 PMC7093122

[19] Duhring S, Germerodt S, Skerka C, Zipfel PF, Dandekar T, Schuster S. 2015. Host-pathogen interactions between the human innate immune system and *Candida albicans*-understanding and modeling defense and evasion strategies. Front Microbiol 6:625. doi:10.3389/fmicb.2015.00625. 26175718 PMC4485224

[20] Zipfel PF, Skerka C. 2009. Complement regulators and inhibitory proteins. Nat Rev Immunol 9:729–740. doi:10.1038/nri2620. 19730437

[21] Fradin C, De Groot P, MacCallum D, Schaller M, Klis F, Odds FC, Hube B. 2005. Granulocytes govern the transcriptional response, morphology and proliferation of *Candida albicans* in human blood. Mol Microbiol 56:397–415. doi:10.1111/j.1365-2958.2005.04557.x. 15813733

[22] Hunniger K, Lehnert T, Bieber K, Martin R, Figge MT, Kurzai O. 2014. A virtual infection model quantifies innate effector mechanisms and *Candida albicans* immune escape in human blood. PLoS Comput Biol 10:e1003479. doi:10.1371/journal.pcbi.1003479. 24586131 PMC3930496

[23] Ding X, Liu Z, Su J, Yan D. 2014. Human serum inhibits adhesion and biofilm formation in *Candida albicans*. BMC Microbiol 14:80. doi:10.1186/1471-2180-14-80. 24673895 PMC4101872

[24] Samaranayake YH, Cheung BP, Yau JY, Yeung SK, Samaranayake LP. 2013. Human serum promotes *Candida albicans* biofilm growth and virulence gene expression on silicone biomaterial. PLoS One 8:e62902. doi:10.1371/journal.pone.0062902. 23704884 PMC3660551

[25] Wachtler B, Citiulo F, Jablonowski N, Forster S, Dalle F, Schaller M, Wilson D, Hube B. 2012. *Candida albicans*-epithelial interactions: dissecting the roles of active penetration, induced endocytosis, and host factors on the infection process. PLoS One 7:e36952. doi:10.1371/journal.pone.0036952. 22606314 PMC3351431

[26] Feng Q, Summers E, Guo B, Fink G. 1999. Ras signaling is required for serum-induced hyphal differentiation in *Candida albicans*. J Bacteriol 181:6339–6346. doi:10.1128/JB.181.20.6339-6346.1999. 10515923 PMC103768

[27] Allert S, Forster TM, Svensson CM, Richardson JP, Pawlik T, Hebecker B, Rudolphi S, Juraschitz M, Schaller M, Blagojevic M, Morschhauser J, Figge MT, Jacobsen ID, Naglik JR, Kasper L, Mogavero S, Hube B. 2018. *Candida albicans*-induced epithelial damage mediates translocation through intestinal barriers. mBio 9:e00915-18. doi:10.1128/mBio.00915-18. 29871918 PMC5989070

[28] Graf K, Last A, Gratz R, Allert S, Linde S, Westermann M, Groger M, Mosig AS, Gresnigt MS, Hube B. 2019. Keeping *Candida* commensal: how lactobacilli antagonize pathogenicity of *Candida albicans* in an *in vitro* gut model. Dis Model Mech 12:dmm039719. 31413153 10.1242/dmm.039719 PMC6765188

[29] Pinsky M, Roy U, Moshe S, Weissman Z, Kornitzer D. 2020. Human serum albumin facilitates heme-iron utilization by fungi. mBio 11:e00607-20. doi:10.1128/mBio.00607-20. 32317324 PMC7175094

[30] Moyes DL, Wilson D, Richardson JP, Mogavero S, Tang SX, Wernecke J, Hofs S, Gratacap RL, Robbins J, Runglall M, Murciano C, Blagojevic M, Thavaraj S, Forster TM, Hebecker B, Kasper L, Vizcay G, Iancu SI, Kichik N, Hader A, Kurzai O, Luo T, Kruger T, Kniemeyer O, Cota E, Bader O, Wheeler RT, Gutsmann T, Hube B, Naglik JR. 2016. Candidalysin is a fungal peptide toxin critical for mucosal infection. Nature 532:64–68. doi:10.1038/nature17625. 27027296 PMC4851236

[31] Wilson D, Naglik JR, Hube B. 2016. The missing link between *Candida albicans* hyphal morphogenesis and host cell damage. PLoS Pathog 12:e1005867. doi:10.1371/journal.ppat.1005867. 27764260 PMC5072684

[32] Mitchell AP. 2016. Microbiology: fungus produces a toxic surprise. Nature 532:41–42. doi:10.1038/nature17319. 27027285

[33] Haeri HH, Schunk B, Tomaszewski J, Schimm H, Gelos MJ, Hinderberger D. 2019. Fatty acid binding to human serum albumin in blood serum characterized by EPR spectroscopy. ChemistryOpen 8:650–656. doi:10.1002/open.201900113. 31143562 PMC6532450

[34] Richardson JP, Mogavero S, Moyes DL, Blagojevic M, Kruger T, Verma AH, Coleman BM, De La Cruz Diaz J, Schulz D, Ponde NO, Carrano G, Kniemeyer O, Wilson D, Bader O, Enoiu SI, Ho J, Kichik N, Gaffen SL, Hube B, Naglik JR. 2018. Processing of *Candida albicans* Ece1p is critical for candidalysin maturation and fungal virulence. mBio 9:e02178-17. doi:10.1128/mBio.02178-17. 29362237 PMC5784256

[35] Takehara K, Yuki K, Shirasawa M, Yamasaki S, Yamada S. 2009. Binding properties of hydrophobic molecules to human serum albumin studied by fluorescence titration. Anal Sci 25:115–120. doi:10.2116/analsci.25.115. 19139584

[36] Swidergall M, Khalaji M, Solis NV, Moyes DL, Drummond RA, Hube B, Lionakis MS, Murdoch C, Filler SG, Naglik JR. 2019. Candidalysin is required for neutrophil recruitment and virulence during systemic *Candida albicans* infection. J Infect Dis 220:1477–1488. doi:10.1093/infdis/jiz322. 31401652 PMC6761979

[37] Richardson JPW, Willems HME, Moyes DL, Shoaie S, Barker KS, Tan SL, Palmer GE, Hube B, Naglik JR, Peters BM. 2018. Candidalysin drives epithelial signaling, neutrophil recruitment, and immunopathology at the vaginal mucosa. Infect Immun 86:e00645-17. doi:10.1128/IAI.00645-17. 29109176 PMC5778364

[38] Henskens YMC, Velden U, Veerman ECI, Amerongen AVN. 1993. Protein, albumin and cystatin concentrations in saliva of healthy subjects and of patients with gingivitis or periodontitis. J Periodontal Res 28:43–48. doi:10.1111/j.1600-0765.1993.tb01049.x. 8426281

[39] Tang LJ, De Seta F, Odreman F, Venge P, Piva C, Guaschino S, Garcia RC. 2007. Proteomic analysis of human cervical-vaginal fluids. J Proteome Res 6:2874–2883. doi:10.1021/pr0700899. 17539673

[40] Raffi RO, Moghissi KS, Sacco AG. 1977. Proteins of human vaginal fluid. Fertil Steril 28:1345–1348. doi:10.1016/s0015-0282(16)42982-1. 590545

[41] Vita GM, De Simone G, Leboffe L, Montagnani F, Mariotti D, Di BS, Luzzati R, Gori A, Ascenzi P, di Masi A. 2020. Human serum albumin binds streptolysin O (SLO) toxin produced by group A *Streptococcus* and inhibits its cytotoxic and hemolytic effects. Front Immunol 11:507092. doi:10.3389/fimmu.2020.507092. 33363530 PMC7752801

[42] Arzumanyan VG, Ozhovan IM, Svitich OA. 2019. Antimicrobial effect of albumin on bacteria and yeast cells. Bull Exp Biol Med 167:763–766. doi:10.1007/s10517-019-04618-6. 31655997

[43] Roy U, Kornitzer D. 2019. Heme-iron acquisition in fungi. Curr Opin Microbiol 52:77–83. doi:10.1016/j.mib.2019.05.006. 31265986

[44] Hube B, Ruchel R, Monod M, Sanglard D, Odds FC. 1998. Functional aspects of secreted *Candida* proteinases. Adv Exp Med Biol 436:339–344. doi:10.1007/978-1-4615-5373-1_47. 9561239

[45] Naglik JR, Challacombe SJ, Hube B. 2003. *Candida albicans* secreted aspartyl proteinases in virulence and pathogenesis. Microbiol Mol Biol Rev 67:400–428. doi:10.1128/MMBR.67.3.400-428.2003. 12966142 PMC193873

[46] Martinez P, Ljungdahl PO. 2005. Divergence of Stp1 and Stp2 transcription factors in *Candida albicans* places virulence factors required for proper nutrient acquisition under amino acid control. Mol Cell Biol 25:9435–9446. doi:10.1128/MCB.25.21.9435-9446.2005. 16227594 PMC1265835

[47] Huber R, Carrell RW. 1989. Implications of the three-dimensional structure of α1-antitrypsin for structure and function of serpins. Biochemistry 28:8951–8966. doi:10.1021/bi00449a001. 2690952

[48] Aggarwal N, Korenbaum E, Mahadeva R, Immenschuh S, Grau V, Dinarello CA, Welte T, Janciauskiene S. 2016. α-Linoleic acid enhances the capacity of α-1 antitrypsin to inhibit lipopolysaccharide induced IL-1β in human blood neutrophils. Mol Med 22:680–693. doi:10.2119/molmed.2016.00119. 27452044 PMC5135082

[49] Frenzel E, Wrenger S, Brugger B, Salipalli S, Immenschuh S, Aggarwal N, Lichtinghagen R, Mahadeva R, Marcondes AM, Dinarello CA, Welte T, Janciauskiene S. 2015. α1-Antitrypsin combines with plasma fatty acids and induces angiopoietin-like protein 4 expression. J Immunol 195:3605–3616. doi:10.4049/jimmunol.1500740. 26363050 PMC6232844

[50] Yumoto R, Suzuka S, Oda K, Nagai J, Takano M. 2012. Endocytic uptake of FITC-albumin by human alveolar epithelial cell line A549. Drug Metab Pharmacokinet 27:336–343. doi:10.2133/dmpk.dmpk-11-rg-127. 22214936

[51] Spellberg B, Ibrahim AS, Edwards JE, Jr, Filler SG. 2005. Mice with disseminated candidiasis die of progressive sepsis. J Infect Dis 192:336–343. doi:10.1086/430952. 15962230

[52] Giles S, Czuprynski C. 2003. Novel role for albumin in innate immunity: serum albumin inhibits the growth of *Blastomyces dermatitidis* yeast form *in vitro*. Infect Immun 71:6648–6652. doi:10.1128/IAI.71.11.6648-6652.2003. 14573690 PMC219601

[53] Wolf JM, Rivera J, Casadevall A. 2012. Serum albumin disrupts *Cryptococcus neoformans* and *Bacillus anthracis* extracellular vesicles. Cell Microbiol 14:762–773. doi:10.1111/j.1462-5822.2012.01757.x. 22289081

[54] Ioannou P, Andrianaki A, Akoumianaki T, Kyrmizi I, Albert N, Perlin D, Samonis G, Kontoyiannis DP, Chamilos G. 2016. Albumin enhances caspofungin activity against *Aspergillus* species by facilitating drug delivery to germinating hyphae. Antimicrob Agents Chemother 60:1226–1233. doi:10.1128/AAC.02026-15. PMC4775923 26643329

[55] Rodrigues AG, Araujo R, Pina-Vaz C. 2005. Human albumin promotes germination, hyphal growth, and antifungal resistance by *Aspergillus fumigatus*. Med Mycol 43:711–717. doi:10.1080/13693780500129814. 16422301

[56] Egesten A, Frick IM, Morgelin M, Olin AI, Bjorck L. 2011. Binding of albumin promotes bacterial survival at the epithelial surface. J Biol Chem 286:2469–2476. doi:10.1074/jbc.M110.148171. 21098039 PMC3024741

[57] Quinn B, Rodman N, Jara E, Fernandez JS, Martinez J, Traglia GM, Montana S, Cantera V, Place K, Bonomo RA, Iriarte A, Ramirez MS. 2018. Human serum albumin alters specific genes that can play a role in survival and persistence in *Acinetobacter baumannii*. Sci Rep 8:14741. doi:10.1038/s41598-018-33072-z. 30282985 PMC6170387

[58] Quinn B, Traglia GM, Nguyen M, Martinez J, Liu C, Fernandez JS, Ramirez MS. 2019. Effect of host human products on natural transformation in *Acinetobacter baumannii*. Curr Microbiol 76:950–953. doi:10.1007/s00284-017-1417-5. 29332139 PMC6890376

[59] Smith AC, Rice A, Sutton B, Gabrilska R, Wessel AK, Whiteley M, Rumbaugh KP. 2017. Albumin inhibits *Pseudomonas aeruginosa* quorum sensing and alters polymicrobial interactions. Infect Immun 85:e00116-17. doi:10.1128/IAI.00116-17. 28630071 PMC5563583

[60] di Masi A, Leboffe L, Polticelli F, Tonon F, Zennaro C, Caterino M, Stano P, Fischer S, Hagele M, Muller M, Kleger A, Papatheodorou P, Nocca G, Arcovito A, Gori A, Ruoppolo M, Barth H, Petrosillo N, Ascenzi P, Di Bella S. 2018. Human serum albumin is an essential component of the host defense mechanism against *Clostridium difficile* intoxication. J Infect Dis 218:1424–1435. doi:10.1093/infdis/jiy338. 29868851

[61] Fulks M, Stout RL, Dolan VF. 2010. Albumin and all-cause mortality risk in insurance applicants. J Insur Med 42:11–17. 21290995

[62] Jellinge ME, Henriksen DP, Hallas P, Brabrand M. 2014. Hypoalbuminemia is a strong predictor of 30-day all-cause mortality in acutely admitted medical patients: a prospective, observational, cohort study. PLoS One 9:e105983. doi:10.1371/journal.pone.0105983. 25148079 PMC4141840

[63] Akirov A, Masri-Iraqi H, Atamna A, Shimon I. 2017. Low albumin levels are associated with mortality risk in hospitalized patients. Am J Med 130:1465e11–1465e19. doi:10.1016/j.amjmed.2017.07.020. 28803138

[64] Moshage HJJ, Janssen JA, Franssen JH, Hafkenscheid JCM, Yap SH. 1987. Study of the molecular mechanism of decreased liver synthesis of albumin in inflammation. J Clin Invest 79:1635–1641. doi:10.1172/JCI113000. 3584463 PMC424488

[65] Gabay C, Kushner I. 1999. Acute-phase proteins and other systemic responses to inflammation. N Engl J Med 340:448–454. doi:10.1056/NEJM199902113400607. 9971870

[66] Soeters PB, Wolfe RR, Shenkin A. 2019. Hypoalbuminemia: pathogenesis and clinical significance. JPEN J Parenter Enteral Nutr 43:181–193. doi:10.1002/jpen.1451. 30288759 PMC7379941

[67] Dominguez de Villota E, Mosquera JM, Rubio JJ, Galdos P, Diez Balda V, de la Serna JL, Tomas MI. 1980. Association of a low serum albumin with infection and increased mortality in critically ill patients. Intensive Care Med 7:19–22. doi:10.1007/BF01692917. 7451716

[68] Minatoguchi S, Nomura A, Imaizumi T, Sasaki S, Ozeki T, Uchida D, Kawarazaki H, Sasai F, Tomita K, Shimizu H, Fujita Y. 2018. Low serum albumin as a risk factor for infection-related in-hospital death among hemodialysis patients hospitalized on suspicion of infectious disease: a Japanese multicenter retrospective cohort study. Ren Replace Ther 4. doi:10.1186/s41100-018-0173-8.

[69] Lee HY, Kang HS, Lee HY, Rhee CK, Lee SY, Kim SC, Kim SJ, Park YJ, Kim YK, Kang JY. 2017. Clinical significance of positive *Pneumocystis jirovecii* polymerase chain reaction in non-human immunodeficiency virus immunocompromised patients in a real practice. Korean J Intern Med 32:478–485. doi:10.3904/kjim.2015.340. 27951623 PMC5432796

[70] Pratikaki M, Platsouka E, Sotiropoulou C, Douka E, Paramythiotou E, Kaltsas P, Kotanidou A, Paniara O, Roussos C, Routsi C. 2011. Epidemiology, risk factors for and outcome of candidaemia among non-neutropenic patients in a Greek intensive care unit. Mycoses 54:154–161. doi:10.1111/j.1439-0507.2009.01787.x. 19793354

[71] Ram R, Swarnalatha G, Neela P, Murty KV. 2008. Fungal peritonitis in patients on continuous ambulatory peritoneal dialysis: a single-centre experience in India. Nephron Clin Pract 110:c207–c212. doi:10.1159/000167867. 18974651

[72] Ogle CK, Wesley Alexander J, Macmillan BG. 1981. The relationship of bacteremia to levels of transferrin, albumin, and total serum protein in burn patients. Burns 8:32–38. doi:10.1016/0305-4179(81)90087-5.

[73] Vogel I, Thorsen P, Flyvbjerg A, Grønbaek H. 2003. Albumin in vaginal fluid is a marker of infection in early pregnancy. Int J Gynaecol Obstet 83:307–308. doi:10.1016/s0020-7292(03)00268-6. 14643045

[74] Corcione S, Angilletta R, Raviolo S, Filippini C, Fossati L, Di Perri G, Cavallo R, De Rosa FG. 2018. Epidemiology and risk factors for mortality in bloodstream infection by CP-Kp, ESBL-E, *Candida* and CDI: a single center retrospective study. Eur J Intern Med 48:44–49. doi:10.1016/j.ejim.2017.10.015. 29096992

[75] Minchiotti L, Caridi G, Campagnoli M, Lugani F, Galliano M, Kragh-Hansen U. 2019. Diagnosis, phenotype, and molecular genetics of congenital analbuminemia. Front Genet 10:336. doi:10.3389/fgene.2019.00336. 31057599 PMC6478806

[76] Gillum AM, Tsay EY, Kirsch DR. 1984. Isolation of the *Candida albicans* gene for orotidine-5′-phosphate decarboxylase by complementation of S. cerevisiae ura3 and *Escherichia coli pyrF* mutations. Mol Gen Genet 198:179–182. doi:10.1007/BF00328721. 6394964

[77] Zakikhany K, Naglik JR, Schmidt-Westhausen A, Holland G, Schaller M, Hube B. 2007. *In vivo* transcript profiling of *Candida albicans* identifies a gene essential for interepithelial dissemination. Cell Microbiol 9:2938–2954. doi:10.1111/j.1462-5822.2007.01009.x. 17645752

[78] Kasper L, König A, Koenig P-A, Gresnigt MS, Westman J, Drummond RA, Lionakis MS, Gross O, Ruland J, Naglik JR, Hube B. 2018. The fungal peptide toxin candidalysin activates the NLRP3 inflammasome and causes cytolysis in mononuclear phagocytes. Nat Commun 9:4260. doi:10.1038/s41467-018-06607-1. 30323213 PMC6189146

[79] Schindelin J, Arganda-Carreras I, Frise E, Kaynig V, Longair M, Pietzsch T, Preibisch S, Rueden C, Saalfeld S, Schmid B, Tinevez JY, White DJ, Hartenstein V, Eliceiri K, Tomancak P, Cardona A. 2012. Fiji: an open-source platform for biological-image analysis. Nat Methods 9:676–682. doi:10.1038/nmeth.2019. 22743772 PMC3855844

[80] Rodrigues DF, Pires das Neves R, Carvalho ATP, Lourdes Bastos M, Costa VM, Carvalho F. 2020. *In vitro* mechanistic studies on alpha-amanitin and its putative antidotes. Arch Toxicol 94:2061–2078. doi:10.1007/s00204-020-02718-1. 32193566

[81] Yang J, Zhang Y. 2015. I-TASSER server: new development for protein structure and function predictions. Nucleic Acids Res 43:W174–W181. doi:10.1093/nar/gkv342. 25883148 PMC4489253

[82] Sugio S, Kashima A, Mochizuki S, Noda M, Kobayashi K. 1999. Crystal structure of human serum albumin at 2.5-Å resolution. Protein Eng 12:439–446. doi:10.1093/protein/12.6.439. 10388840

[83] Ramirez-Aportela E, Lopez-Blanco JR, Chacon P. 2016. FRODOCK 2.0: fast protein-protein docking server. Bioinformatics 32:2386–2388. 27153583 10.1093/bioinformatics/btw141

