# Supplementary figures and images for: Albumin Neutralizes Hydrophobic Toxins and Modulates Candida albicans Pathogenicity
Source: mBio. 2021 Jun 22;12(3):e00531-21. doi: 10.1128/mBio.00531-21 (PMC8262992; doi:10.1128/mBio.00531-21)

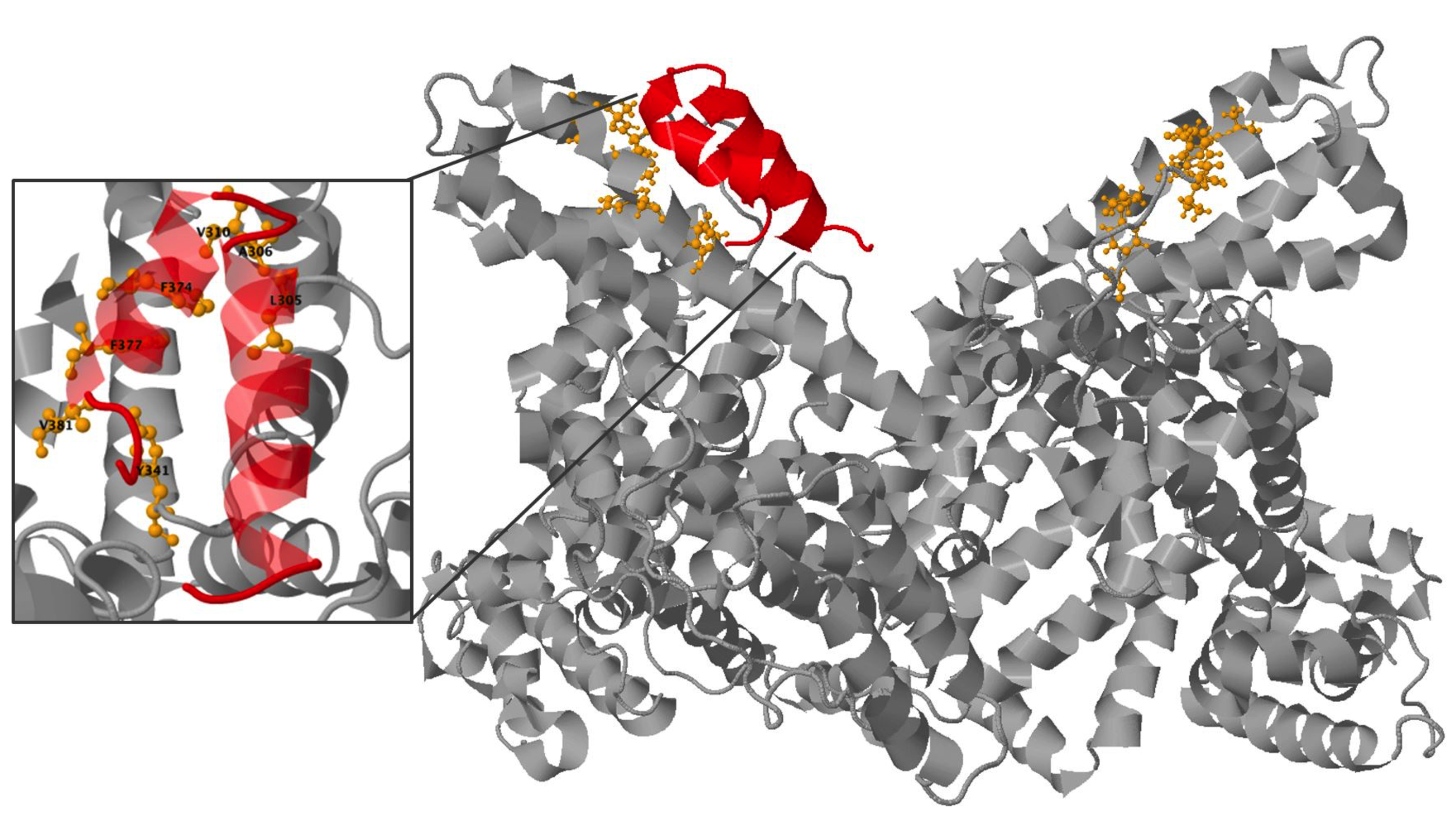

Supplement: FIG S1 [file mbio.00531-21-sf001.tif]
